# Lamprey *FOXN1* rescues the block of thymic epithelial cell development in the mouse *Foxn1*-deficient thymic rudiment

**DOI:** 10.1073/pnas.2520664122

**Published:** 2025-11-25

**Authors:** Ryo Morimoto, Gaoqun Zhang, Oliver S. Thomas, Margaret F. Docker, Jonah L. Yick, Floriaan Devloo-Delva, Jeremy Swann, Dagmar Diekhoff, Thomas Boehm

**Affiliations:** ^a^Max Planck Institute of Immunobiology and Epigenetics, Freiburg D-79108, Germany; ^b^Max Planck Institute for Biology Tuebingen, Tuebingen D-72076, Germany; ^c^Department of Biological Sciences, University of Manitoba, Winnipeg, MB RT3 2N2, Canada; ^d^Inland Fisheries Service, New Norfolk, TAS 7140, Australia; ^e^Australian National Fish Collection, National Collections and Marine Infrastructure, Commonwealth Scientific and Industrial Research Organization, Hobart, TAS 7000, Australia; ^f^Institute for Immunodeficiency, Center for Chronic Immunodeficiency, University Medical Center, Freiburg D-79106, Germany

**Keywords:** thymus, mouse, lamprey, Foxn1, lymphocytes

## Abstract

All vertebrate adaptive immune systems exhibit distinct lymphocyte lineages. In jawless vertebrates, such as lampreys, T-like cells are thought to develop in lympho-epithelial structures at the tips of gill filaments, termed thymoids. However, it is unclear whether thymoids are functionally equivalent to the thymus of jawed vertebrates. Indeed, because the structural modules that are somatically assembled to form the antigen receptors of jawless and jawed vertebrates differ, development and selection of T cells may be governed by clade-specific genetic networks. To address this question, we have replaced the mouse *Foxn1* gene, a key regulator of the thymic microenvironment in jawed vertebrates, with the orthologous *FOXN1* lamprey gene, which is expressed in the thymoids alongside genes orthologous to known targets of the mouse Foxn1 transcription factor. The reconstituted thymi support normal T cell development, and, to a lesser extent, also support B cell development, indicating that the lamprey *FOXN1* gene can rescue the block of thymic epithelial cell differentiation in mice deficient for the endogenous *Foxn1* gene. The absence of overt autoimmunity in transgenic mice suggests that the reconstituted thymic microenvironment directs the development of a self-tolerant T cell repertoire. These findings highlight the remarkable similarity of thymic epithelial functions in jawed and jawless vertebrates, despite more than 500 My of independent evolution. Our results thus suggest that the emergence of the Foxn1 transcription factor in the common ancestor of vertebrates was associated with the advent of a specialized tissue environment supporting the development and selection of T cells.

## Significance

Primary lymphoid organs, such as the thymus, are cornerstones of vertebrate immune systems. Thymopoietic tissues have long been recognized in jawed vertebrates, ranging from cartilaginous fishes to humans, and in jawless vertebrates, such as lampreys. However, because the molecular structures of antigen receptors differ between jawed and jawless vertebrates, it has remained unclear whether the intrathymic selection processes that ensure the generation of a self-tolerant repertoire of T cell specificities also have at least some commonalities. We show here that the lamprey *FOXN1* gene can substitute for the mouse *Foxn1* gene to generate a self-tolerant T cell compartment. These findings offer a glimpse into the structure and function of the thymopoietic niche of the ancestor common to all vertebrates.

Substantial modifications of the immune system occurred in early vertebrates. For instance, specialized lymphopoietic tissues emerged to support the development of lymphocytes as a new type of hematopoietic cells (1). The thymus represents one such distinct evolutionary novelty (2), although its function was only recognized in jawed vertebrates about 60 y ago (3–5). In mammals, the thymus is formed through the coalescence of small lobules of cortical and medullary units (6, 7), a developmental scenario that likely applies to all jawed vertebrates. Recently, a thymus candidate has also been recognized in ammocoete larvae of lampreys; the thymoid tissues of lampreys are found in the entire gill basket and are organized as small lobes situated at the tips of gill filaments, each possibly representing such minimal thymic developmental units (8).

In mammals, the thymus generates a self-tolerant repertoire of T cells arising from incoming multipotent hematopoietic progenitors (9). Tolerance is achieved by the expression of peripheral self-antigens in the thymic epithelium, and the presence of small populations of cells mimicking the diverse phenotypes of peripheral tissues (10–13). In the thymus, self-antigens are presented to T cells in complex with major histocompatibility complex (MHC) molecules; the strength of interaction with clonally expressed T cell receptors determines the fate of developing thymocytes, resulting in death by neglect, positive selection, or conversion to a regulatory fate (8, 14). Whereas the development of T cells in jawed vertebrates is well studied, the mechanistic underpinnings of T-like cell development in the thymoid of lampreys and the selection processes shaping the antigen receptor repertoire are largely unexplored (15). The different variable lymphocyte receptors (VLRs) of lampreys (16) are expressed by distinct lymphocyte lineages (16, 17). They are composed of leucine-rich repeat modules (16, 18) that are assembled into functional genes from incomplete germ-line sequences in a gene conversion-like mechanism (18, 19) that is orchestrated by cytidine deaminases (20, 21). The fact that lamprey genomes do not encode MHC molecules (22), and that their antigen receptors are molecularly distinct from those of jawed vertebrates, has led to the hypothesis that the molecular mechanism(s) governing antigen recognition in lampreys are different from those of jawed vertebrates. However, certain aspects of the intrathymic selection process, such as those that pertain to antigen processing, may be shared among jawless and jawed vertebrates.

In mammals, the thymic microenvironment orchestrates the development and selection of T cells; its function depends on the activity of the Foxn1 transcription factor, which directs the differentiation of specific regions of the pharyngeal epithelium to acquire lymphopoietic activity (23–25). Some of the direct target genes of Foxn1 are known (26–30). For instance, the chemokine Cxcl12 directs the migration of immature hematopoietic precursors to the thymic rudiment (28, 31), whereas the Notch ligand Dll4 initiates the T cell differentiation programme (32, 33). Reconstitution experiments in mice have shown that the expression of these two components in the *Foxn1*-deficient thymic rudiment alone is sufficient to support development of CD4^+^CD8^+^ double positive thymocytes (28). Genes encoding components required for antigen processing, such as the thymic proteasome component Psmb11 and the serine protease Prss16, the latter known for its role in the selection of CD4+ cells in mammals (34), also appear to be direct targets of Foxn1 (29). By contrast, additional factors, such as Fezf2 (35) and Aire (36), and components of the antigen presentation pathways themselves (such as MHC, etc.) appear to be positioned further downstream along the Foxn1-initiated differentiation programme. Therefore, additional transcription factors acting downstream of Foxn1 (and/or in parallel) are likely required to generate a fully functional thymic microenvironment. In previous studies, we have found that cells in the thymoid of the jawless vertebrate *Lampetra planeri* express *FOXN1* and *DLL* (8), raising the possibility that all vertebrate Foxn1 transcription factors [and by inference also their evolutionarily ancestral Foxn4 paralogs (37–40)] control the expression of the same core set of genes whose products guide the first steps of T cell development and may even support certain aspects of the antigen processing process.

Here, we set out to further explore the characteristics of thymopoiesis of lampreys. First, we examined the expression of lamprey orthologs of several genes known to play pivotal roles in regulating thymopoiesis in jawed vertebrates (37). Second, to circumvent the complications associated with genetic interference in lampreys, we explored the function of the lamprey *FOXN1* gene in the mammalian context by substituting it for the endogenous mouse *Foxn1* gene. Our results define a common set of genes expressed in thymopoietic tissues of both jawless and jawed vertebrates, and demonstrate that lamprey *FOXN1* genes are capable of restoring T cell development in *Foxn1*-deficient mice. These findings provide strong support for evolutionarily conserved functions of *Foxn1* orthologs in promoting thymopoiesis across the two major branches of vertebrates.

## Results

### Gene Expression Patterns in the Lamprey Thymoid.

**Fig. 1.**
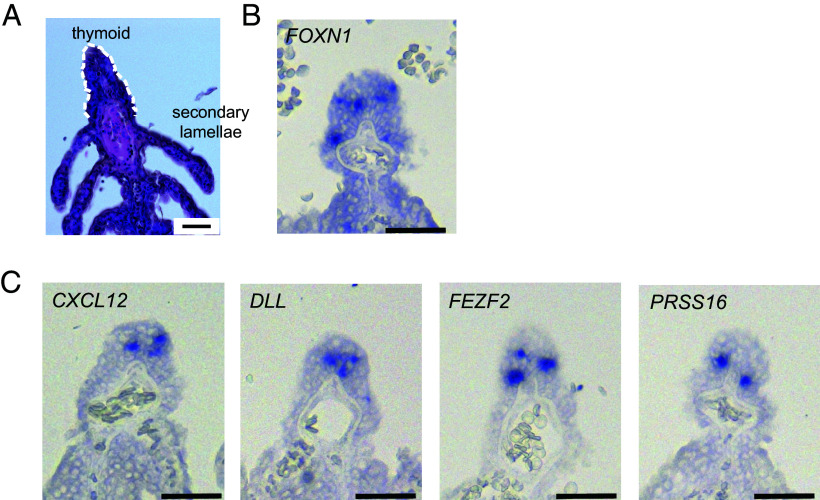
Expression profiles in the lamprey larval thymoid. (*A*) Histological appearance of the thymoid at the tip of gill filaments (*L. planeri*; hematoxylin & eosin staining). (*B* and *C*) RNA in situ hybridization of *L. planeri* thymoid tissues with the indicated gene-specific probes. (Scale bar, 0.05 mm.)

Cells in the thymoid of ammocoete larvae (Fig. 1*A*) of the European brook lamprey *L. planeri* are known to express *FOXN1* (Fig. 1*B*) and one of its key target genes, *DLL* (Fig. 1*C*) (8). To explore the gene expression profile of the thymoid further, we examined the expression of *CXCL12*, encoding a ligand of the Cxcr4 chemokine receptor (41). The *CXCL12*-expressing cells are located in the inner aspect of the thymoid, as are those that express the Notch ligand DLL (Fig. 1*C*). Thus, the minimal set of thymopoietic factors that in mice are required to attract hematopoietic progenitor cells (Cxcl12) and to initiate their differentiation into the T cell lineage (Dll4) (28, 32, 33) are also expressed in the lamprey thymoid. Because the lamprey genome does not contain the gene encoding the autoimmune regulator Aire, we instead examined the expression of *FEZF2*, encoding what might represent the primordial (13) regulator of expression of tissue-restricted antigens in thymopoietic environments (35, 36). Whereas the gene encoding the thymic-specific proteasome component Psmb11 does not exist in lampreys, the Prss16 serine protease is encoded in the lamprey genome (42). Both *FEZF2* and *PRSS16* are expressed in the lamprey thymoid (Fig. 1*C*), indicating that even some components of the intrathymic antigen processing machinery are shared between jawless and jawed vertebrates.

We considered two strategies to provide direct evidence for the role of lamprey *FOXN1* genes in the genetic network underlying thymopoiesis. CRISPR/Cas-mediated genetic inactivation of the lamprey *FOXN1* gene failed as it proved difficult to raise sufficient numbers of *FOXN1* lamprey crispants to the stage when the first T-like cells appear, which typically occurs around 3 to 4 mo after in vitro fertilization (21). In an alternative strategy, we replaced the mouse *Foxn1* gene by its lamprey ortholog to assess its capacity to rescue the mutant phenotype (*SI Appendix*, Fig. S1 *A*–*E*). Here, we report on the results of the latter strategy.

### Thymopoietic Activity of the Lamprey *FOXN1* Gene.

**Fig. 2.**
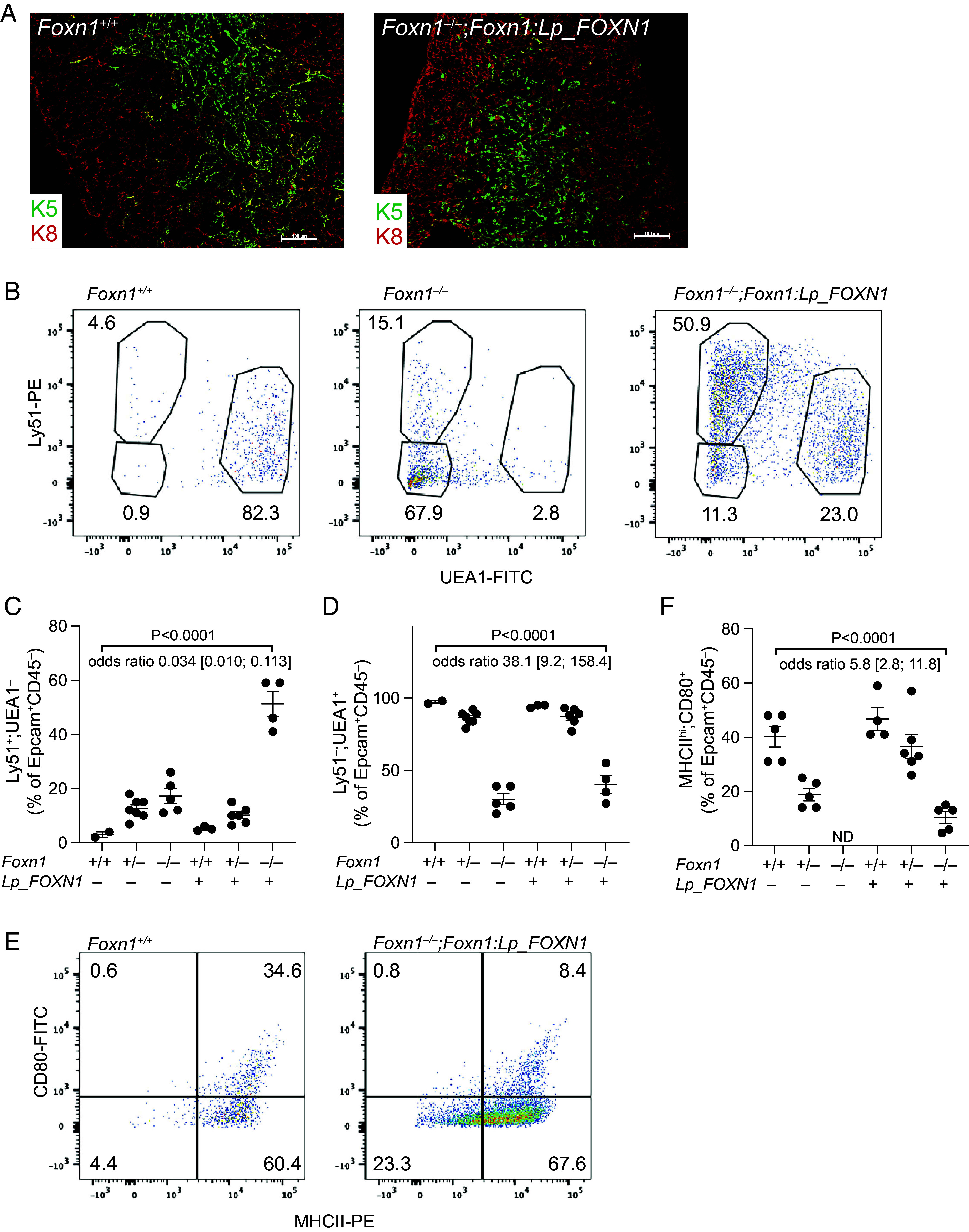
Thymopoietic activity of the *FOXN1* gene of *L. planeri* in *Foxn1*-deficient mice. (*A*) Immunohistology using anti-keratin antibodies of thymus sections of wild type mice (*Foxn1*^+/+^) and transgenic mice (*Foxn1*^–/–^;*Foxn1:Lp_FOXN1*); K5, green; K8, red. (Scale bar, 0.1 mm.) (*B–F*) TEC phenotype of *Foxn1:Lp_FOXN1* transgenic mice. (*B*) Representative flow cytometric profiles for Ly51^+^ and UEA1^+^ TECs for wild type (*Foxn1*^+/+^), *Foxn1*-deficient (*Foxn1*^–/–^), and mice expressing the *L. planeri FOXN1* gene on the *Foxn1*-deficient background (*Foxn1*^–/–^;*Foxn1:Lp_FOXN1*). (*C*) Proportions of TECs with cortical (Ly51^+^;UEA1^–^) phenotype. (*D*) Proportions of TECs with medullary (Ly51^–^;UEA1^+^) phenotypes. (*E*) Representative flow cytometric profiles of TECs stained for CD80 and MHCII surface molecules. (*F*) Proportions of TECs with mature mTEC (MHCII^hi^;CD80^+^) phenotype. ND denotes the absence of cells with the indicated phenotype. For panels (*C*, *D*, and *F*), the sample mean ± SEM is shown. *P* values and odds ratios of proportions for the indicated comparisons are indicated, with 95% CI in square brackets.

**Fig. 3.**
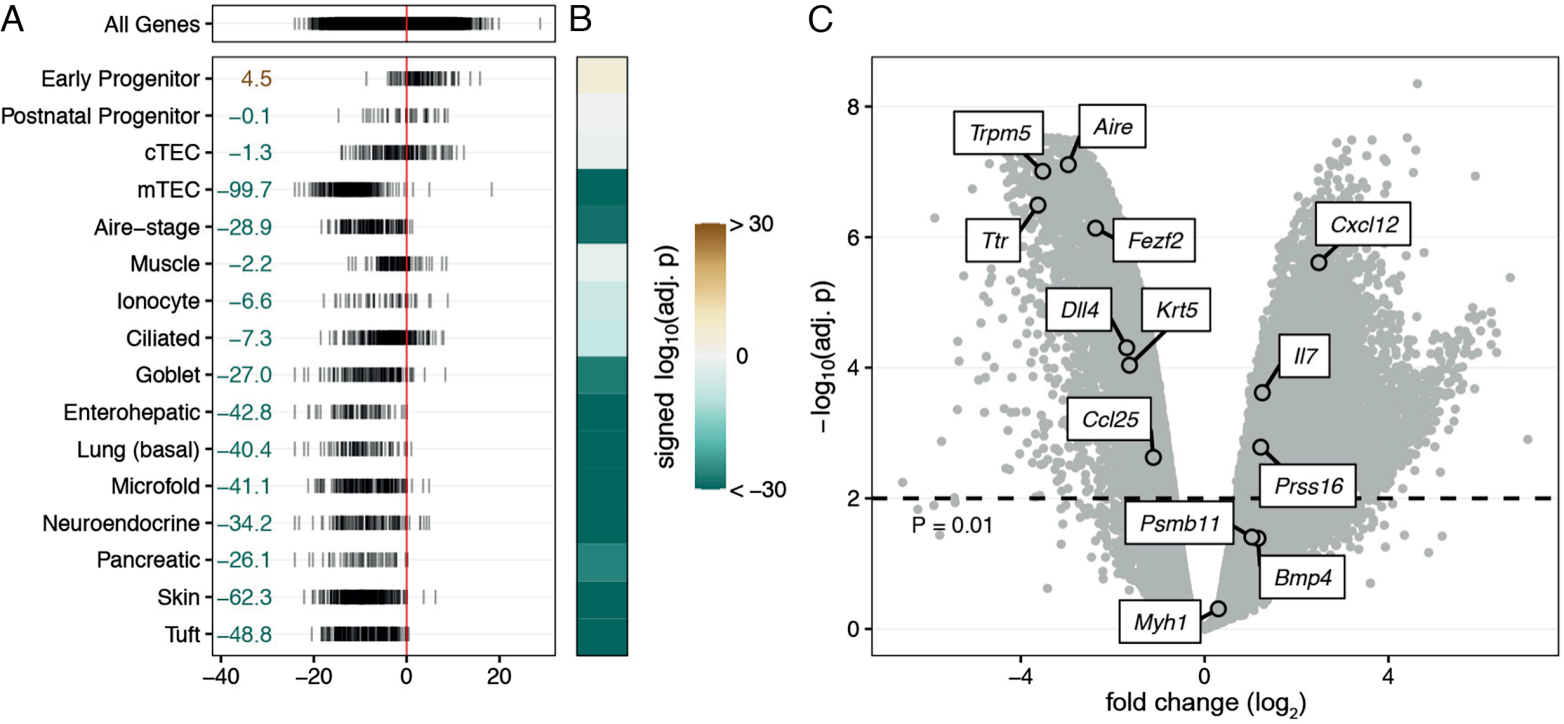
Gene expression changes between TECs in wild type and *L. planeri FOXN1* transgenic mice. (*A*) Overview of expression changes of all genes (*Top*) and genes associated with the indicated TEC signatures (*Bottom*). Each line represents one gene and shows the t-statistic derived from differential expression analysis. Numbers on the left indicate the signed log10(adj. *P*) values from enrichment analysis with *camera*. The sign indicates the direction of change; i.e., positive values indicate enrichment and negative values indicate depletion relative to wild type. (*B*) Log-transformed and signed *P* values of data in (*A*) visualized as a heatmap. Values outside the color scale were clipped to the nearest limit. (*C*) Volcano plot showing the results of differential gene expression analysis. Selected genes of interest are labeled. The analysis is based on 3 wild type and 4 *L. planeri FOXN1* transgenic adult mice.

In mice lacking the *Foxn1* gene, the ability of the thymic rudiment to attract multipotent hematopoietic progenitors and its lymphopoietic and tolerogenic capacities are abolished (23). However, transgenic expression of a functional mouse *Foxn1* gene in the thymic epithelium of *Foxn1*-deficient mice fully restores thymus function (38, 39), establishing an experimental platform to assess the lymphopoietic activities of any member of this transcription factor gene family. We turned to this type of heterologous replacement strategy in order to directly examine the functional capacity of lamprey *FOXN1* genes in the context of the mammalian immune system, which we envisioned would reveal its interspecific target gene activity. The mouse *Foxn1* gene not only controls the differentiation of the thymic epithelium, but also the keratinization of hair shafts (43). Expression of the *Lampetra planeri FOXN1* gene under the control of the transcriptional regulatory elements of the mouse *Foxn1* locus restored the hair coat of *Foxn1*-deficient mice (*SI Appendix*, Fig. S1), providing a convenient marker of transgene activity. Even more surprisingly, expression of the lamprey *FOXN1* gene in the *Foxn1*-deficient thymic epithelium restored lymphopoiesis in the thymic rudiment. Although smaller than the wild type counterpart (*SI Appendix*, Fig. S2*A*), the thymus exhibited clearly demarcated cortical and medullary areas, mirrored in the characteristic keratin 5 and keratin 8 expression pattern (Fig. 2*A*). However, flow cytometric characterization of thymic epithelial cells (TECs) indicates that the proportion of Ly51-positive cortical TEC-like cells is greatly increased, whereas that of UEA-1 positive medullary TECs is diminished (Fig. 2 *B*–*D*); nonetheless, the reconstituted epithelial compartment harbors MHCII^hi^CD80^+^ TECs, a key component of the tolerogenic microenvironment (Fig. 2 *E* and *F*), albeit at reduced frequency. The emergence of Aire-expressing cells in the medullary region indicates that at least some epithelial cells reach a fully mature stage (*SI Appendix*, Fig. S2*B*). To further characterize the transgenic epithelial compartment, we carried out RNAseq analysis on purified TEC populations and performed competitive enrichment analysis of epithelial cell type–specific gene expression signatures (13). Among canonical TEC signatures represented in the lamprey *FOXN1*-expressing epithelium, we observed an enrichment of the early progenitor compartment compared to the mouse wild type microenvironment, whereas the proportion of the postnatal TEC precursor signature is essentially unchanged (Fig. 3 *A* and *B*). Little difference was observed for the cTEC signature; by contrast, a drastic reduction of the mTEC signature is apparent (Fig. 3 *A* and *B*). Collectively, the results of flow cytometry and gene expression analysis suggest that the maturation of the mTEC compartment, although clearly impaired, is not completely blocked. Next, we examined the representation of mimetic cell signatures in the reconstituted thymus. Interestingly, as expected from previous observations (13), the representation of signatures for mimetic cell types whose development does not require Foxn1 transcription factor activity, such as muscle and ciliated cells, is similar to wild type mice (Fig. 3 *A* and *B* and *SI Appendix*, Fig. S3); by contrast, the signatures of *Foxn1*-dependent enterohepatic, microfold, pancreatic, skin, and tuft cells are reduced (Fig. 3 *A* and *B* and *SI Appendix*, Fig. S3), in line with the impaired development of the mTEC compartment (13). Differential gene expression analysis indicates that the expression levels of key genes of tolerogenic mTECs, such as *Aire* and *Fezf2* are reduced; this is also true for genes associated with certain mimetic cells, such as tuft cells (*Trpm5*) and enterohepatic cells (*Ttr*). By contrast, the muscle-specific *Myh1* gene expression level is unchanged (Fig. 3*C*).

To examine the possibility that the altered differentiation of the TEC compartment leads to incomplete central tolerance formation and subsequently to autoimmunity, we followed the transgenic mice until the age of about 1 y. During this time, no obvious clinical signs of autoimmunity, such as premature death, diarrhea, skin lesions, etc. were observed; moreover, histological sections of salivary gland, lung, kidney, small intestine, pancreas, liver, and colon showed no signs of tissue infiltrations (*SI Appendix*, Fig. S4), suggesting that self-tolerance of the emerging T cell repertoire and peripheral immune homeostasis is successfully established.

In sum, these findings suggest that despite substantial sequence variation, particularly notable in the N-terminal regions (*SI Appendix*, Fig. S5), the lamprey FOXN1 transcription factor is capable of successfully initiating the differentiation of TECs, revealing a remarkable level of functional conservation of the regulatory regions of critical Foxn1 target genes over hundreds of millions of years of independent evolution in the jawless and jawed vertebrate branches.

### Lymphopoietic Activity of Lamprey *FOXN1* Genes.

**Fig. 4.**
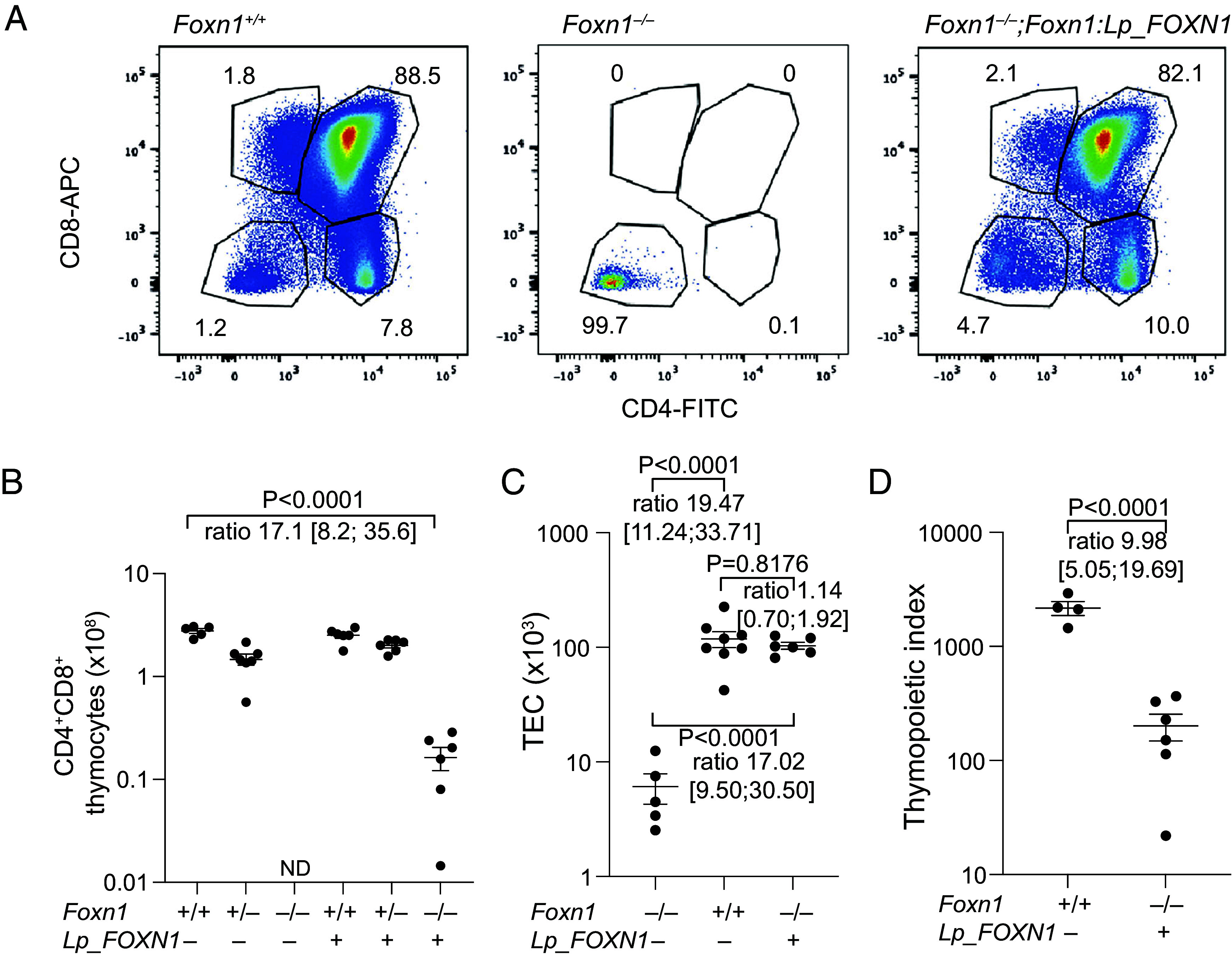
Flow cytometric analyses of thymocytes of *Foxn1*:*Lp_FOXN1* transgenic mice. (*A*) Representative flow cytometric profiles for CD45^+^ cells resolved with anti-CD4 and anti-CD8 antibodies for the indicated genotypes. (*B*) Numerical evaluation of CD4^+^CD8^+^ double-positive thymocytes for the indicated genotypes. (*C*) Numerical evaluation of EpCAM+CD45–TECs for the indicated genotypes. (*D*) Numerical evaluation of the thymopoietic index (ratio of the number of CD4^+^CD8^+^ double-positive thymocytes and TECs) for the indicated genotypes. For panels (*B*–*D*), the sample mean ± SEM is shown. *P* values and estimated ratios of cell numbers for the indicated comparisons are indicated, with 95% CI in square brackets.

Next, we assessed the lymphopoietic activity of the transgene by analysis of the composition of intrathymic hematopoietic cells. The transgenic thymus harbors a substantial number of CD4^+^CD8^+^ double-positive thymocytes (DP) (Fig. 4*A*), reaching approximately 6% of wild-type levels (Fig. 4*B*). However, wild type thymi and transgenic thymi harbor essentially the same numbers of TECs (Fig. 4*C*), indicating that the proliferative burst associated with the development of the thymic microenvironment is supported by the lamprey FOXN1 transcription factor. Nonetheless, the thymopoietic index (ratio of DP thymocytes and TECs) is greatly reduced (Fig. 4*D*), suggesting that the TEC population harbors a substantial fraction of cells with reduced thymopoietic capacity, in line with the greater preponderance of progenitor cells (Fig. 3). The CD4^+^/CD8^+^ thymocyte ratio is similar between wild type mice and the transgenic mice [ratio of sample means, 4.15 vs. 4.43 (*SI Appendix*, Fig. S6 *A* and *B*)], indicating that the mature component of the TEC compartment is functionally indistinguishable from its wild type counterpart. Interestingly, codominant expression of the endogenous *Foxn1* gene and the Lp_*FOXN1* transgene results in T cell numbers similar to their nontransgenic siblings (Fig. 4*B* and *SI Appendix*, Fig. S6); by contrast, the TEC-specific expression of the *FOXN4* gene of the cephalochordate *Branchiostoma lanceolatum* (representing one of the nonvertebrate ancestors of vertebrate *Foxn1* genes) (39) alongside the endogenous mouse *Foxn1* gene is associated with a marked reduction of T cell poiesis, indicative of a strong dominant-negative effect (*SI Appendix*, Fig. S6 *C* and *D*). This discrepancy can be explained by a restricted target gene range of the *B. lanceolatum* FOXN4 transcription factor, beyond those encoding a chemokine and a Notch ligand. Conversely, the lack of appreciable dominant-negative activity of the lamprey *FOXN1* gene when expressed together with the endogenous mouse *Foxn1* gene indicates that it is functionally similar to the endogenous mouse Foxn1 transcription factor.

The reduction of thymocyte numbers in the transgenic “lampetroid” thymus most likely results from inefficient intrathymic differentiation of developing T cells, for instance reflected in an increased ratio of DN1 and DN3 cells (*SI Appendix*, Fig. S6 *E*–*G*), presumably owing to the reduced expansion of DN3 thymocytes. In line with the overall diminished thymopoietic capacity of the transgenic thymus, the peripheral compartment exhibits T lymphopenia, albeit with normal ratios of CD4^+^ and CD8^+^ lymphocytes; as a result, the proportion of B cells (non-T cells) in the lymph nodes is increased (*SI Appendix*, Fig. S7), as B cell development in mice is not impaired by *Foxn1* deficiency.

**Fig. 5.**
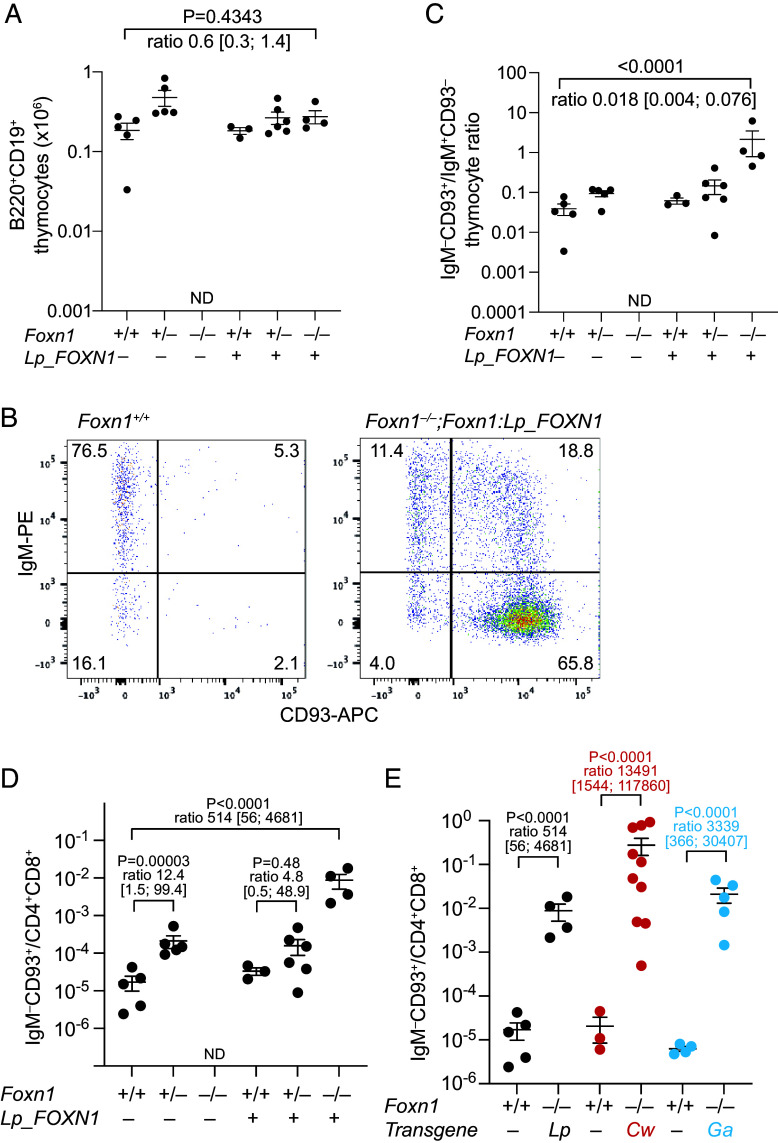
B cell poietic capacity driven by lamprey *FOXN1* genes in mice. (*A*) Numerical evaluation of B220^+^CD19^+^ cells for the indicated genotypes. (*B*) Representative flow cytometric profiles for CD45^+^B220^+^CD19^+^ cells for the indicated genotypes resolved with anti-IgM and anti-CD93 antibodies. (*C*) Ratios of immature (IgM^–^CD93^+^) and mature (IgM^+^CD93^–^) B cells in the thymus for the indicated genotypes. (*D*) Ratios of immature (IgM^–^CD93^+^) B cells and immature (CD4^+^CD8^+^) T cells in the thymus for the indicated genotypes. ND denotes the absence of cells with the indicated phenotype. (*E*) Ratios of immature (IgM^–^CD93^+^) B cells and immature (CD4^+^CD8^+^) T cells in the thymus of *Foxn1*-deficient mice transgenic for three lamprey *FOXN1* genes. Lp, *L. planeri*; Cw, *Caspiomyzon wagneri*; Ga, *Geotria australis*. ND denotes the absence of cells with the indicated phenotype. For panels (*B*–*D*), the sample mean ± SEM is shown. *P* values and estimated ratios of cell numbers for the indicated comparisons are indicated, with 95% CI in square brackets.

Despite significantly reduced numbers of CD4^+^CD8^+^ double-positive thymocytes, the number of B220^+^CD19^+^ B cells in the CD4^–^CD8^–^ double-negative fraction of the *Lp_FOXN1* transgenic thymus remains unchanged (Fig. 5*A*), indicating a shift toward a higher B cell fraction. Moreover, the B cells in the transgenic thymus are predominantly immature, as assessed by the increased ratio of immature (IgM^–^CD93^+^) to mature (IgM^+^CD93^–^) cells (Fig. 5 *B* and *C*). The imbalance of T and B cell development in the transgenic thymi is accompanied by altered expression levels of *Dll4* and *Il-7* genes in the reconstituted thymus (Fig. 3*C*). We have previously shown that reduced levels of *Dll4* gene expression together with higher *Il-7* gene activity results in a B cell supportive thymic microenvironment (39). Hence, the phenotypic consequences of altered gene expression patterns support the notion that B cell poiesis becomes more prominent when the expression levels of T cell–inducing factors, most notably Dll4, a key target gene of mouse Foxn1, are limiting (28, 32, 33). We confirmed the observed thymopoietic characteristics of lamprey *FOXN1* orthologs by constructing additional transgenic lines expressing *FOXN1* genes of *Caspiomyzon wagneri* (Caspian lamprey; *Cw_FOXN1*), and *Geotria australis* (pouched lamprey; *Ga_FOXN1*). In these transgenic lines, T cells develop normally (*SI Appendix*, Fig. S8), albeit in reduced numbers, as seen also for the restoration driven by the *FOXN1* gene of *L. planeri* (Fig. 4). In sum, our results indicate that even evolutionarily distantly related vertebrate *Foxn1* genes are capable of supporting the differentiation of mature T cells in the mouse thymus, albeit with reduced efficiencies when compared to the endogenous gene.

### Intrathymic T Cell and B Cell Development.

The presence of immature B cells in the thymus reconstituted with the *L. planeri FOXN1* gene prompted us to examine this phenomenon also in the two other lamprey transgenic lines. We noted that in nontransgenic mice, *Foxn1* haploinsufficiency increases the ratio of IgM^–^CD93^+^ immature B cells to CD4^+^CD8^+^ double-positive thymocytes by an order of magnitude, as a result of reduced numbers of CD4^+^CD8^+^ double-positive thymocytes and increased numbers of IgM^–^CD93^+^ immature B cells (29) (Fig. 5*D*). In the thymus reconstituted with *Lp_FOXN1*, the lymphopoietic shift toward B cell poiesis is more pronounced [with a ~ 500-fold increase (Fig. 5*D*)]; the corresponding shifts for the *Cw_FOXN1* and *Ga_FOXN1* transgenic constellations are similarly drastic (by several orders of magnitude) (Fig. 5*E*). Additional experiments are required to determine whether the increased B cell compartment arises from a T-to-B cell conversion, or whether the lamprey *FOXN1*-driven thymic epithelium provides a niche supporting extrathymically derived B cell precursors (44). Nonetheless, despite their increased B cell signature, all three reconstituted lampetroid thymi favor T cell over B cell development. Of note, the thymopoietic characteristics of lamprey *FOXN1* orthologs are more similar to that of the elephant shark (*Callorhinchus milii*) *Foxn4* than to that of the paralogous *Foxn1* gene of *C. milii* (39). Indeed, sequence comparisons suggest a closer relationship of the lamprey *FOXN1* genes with the Foxn4 gene clade of jawed vertebrates, most notably with respect to residues of the DNA binding domain (*SI Appendix*, Fig. S5 *B* and *C*).

### B Cell Development in the Lamprey Thymoid.

**Fig. 6.**
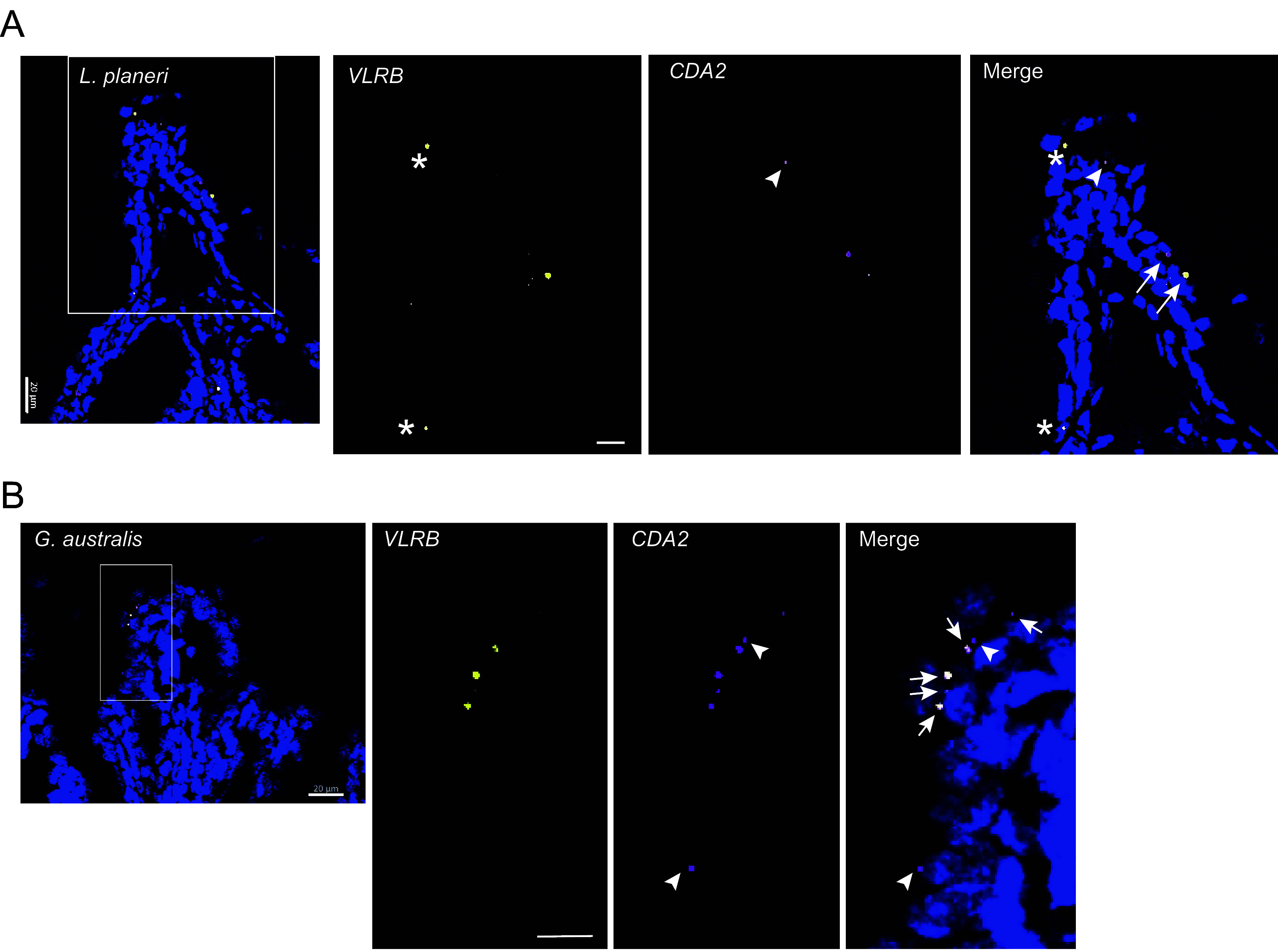
B cells in the lamprey thymoid region. (*A*) RNA in situ hybridization of *L. planeri* thymoid tissues with probes specific for *VLRB* and *CDA2*. The leftmost panel provides an overview (blue: DAPI staining; scale bar, 0.02 mm) indicating the region shown at higher power in the *Right* panels (scale bar, 0.01 mm); the rightmost panels are a superposition of the two middle panels (asterisk, *VLRB* single-positive cells; arrowheads, *CDA2* single-positive cells; arrows, *VLRB/CDA2* double-positive cells). The *VLRB* probe was haptenized with fluorescein, the *CDA2* probe was haptenized with DIG. (*B*) RNA in situ hybridization of *G. australis* thymoid tissues with probes specific for *VLRB* and *CDA2*. Designations and scale bars are as in (*A*). The *VLRB* probe was haptenized with DIG, the *CDA2* probe was haptenized with fluorescein.

Given the augmented B cell supportive nature of lymphopoiesis in the reconstituted mouse thymi, we next asked whether the B cell poietic activity of lamprey *FOXN1* genes may also be detectable in the thymoid of ammocoete larvae. In previous work, we have shown that the thymoid is characterized by a large accumulation of cytidine deaminase 1 (*CDA1*)-positive cells in its outer (“cortical”) region, presumed to reflect the ongoing assembly in immature thymocytes of T-lineage related *VLR* genes (such as, *VLRA* and *VLRC*) by CDA1; by contrast, *VLRB*-expressing and *CDA2*-expressing cells are abundant in the typhlosole of ammocoete larvae as the main site of B cell differentiation in the larva (8). Using a sensitive double-labeling RNA in situ hybridization technique, we searched for rare *VLRB*-expressing cells in the thymoid. We used a *VLRB*-specific probe, which reveals transcriptional activity at the *VLRB* locus without regard to its assembly status; in addition, we used a *CDA2*-specific probe, recording the expression of the B lymphocyte-lineage-specific cytidine deaminase gene that is required for *VLRB* gene assembly (21). Given the lack of information on the developmental trajectory of lamprey B cell development, we focused our attention on the presence of *CDA2*^+^*VLRB*^+^ double positive cells in the thymoid, which we presumed to represent an intermediate stage of B cell development. Using this sensitive assay, *CDA2*^+^*VLRB*^+^ double positive cells were indeed detected; they are invariably located in the cortical region of the thymoids of *L. planeri* (Fig. 6*A*) and *G. australis* (Fig. 6*B*). The *CDA2*^+^*VLRB*^–^ cells, also found predominantly in the cortex, might represent B lineage cells in earlier stages of development. In summary, the analysis of the lymphopoietic signature of the larval thymoid qualitatively reflects the outcome of the reconstitution experiments in the mammalian context.

## Discussion

Early in vertebrate evolution, adaptive immune facilities emerged at least twice. Characteristic innovations encompass dedicated primary lymphoid tissues, genome editors to generate functional antigen receptors, and functionally distinct lymphocyte lineages with clonal expression of antigen receptors (1). Because the adaptive immune systems of jawless and jawed vertebrates differ with respect to the molecular nature of the antigen receptors and the mechanisms by which they are assembled during lymphocyte development, it is generally assumed that the mechanism(s) underlying the generation and maintenance of self-tolerance are unlikely to be the same in the two branches of vertebrates that separated about 550 Mya (45). Here, we focus on the characterization of the thymoid, the thymus equivalent of lampreys that is presumed to be the site of T cell development (8).

In the gene regulatory network of TEC differentiation, the Foxn1 transcription factor occupies a key position (23–25). Whereas a rich experimental armamentarium to identify target genes of Foxn1 in the mammalian system has been successfully used (26–30), its application to the lamprey system is fraught with a number of difficulties, such as the long generation time of lampreys in the order of five or more years, and the difficulties associated with completing the entire reproductive cycle under laboratory conditions, in particular for parasitic lampreys. Although CRISPR/Cas-mediated mutagenesis can be used to probe immune system function (21), we have so far failed to generate *FOXN1* crispants amenable to meaningful immune system analysis, as they were lost early in development, perhaps owing to the ensuing T cell immunodeficiency and/or pleiotropic effects of *FOXN1* mutations. As an alternative strategy, we probed the function of the lamprey *FOXN1* gene by transferring it to a mammalian immune system. Remarkably, the lamprey *FOXN1* gene was capable of rescuing the athymic phenotype of *Foxn1* deficiency in mice.

**Fig. 7.**
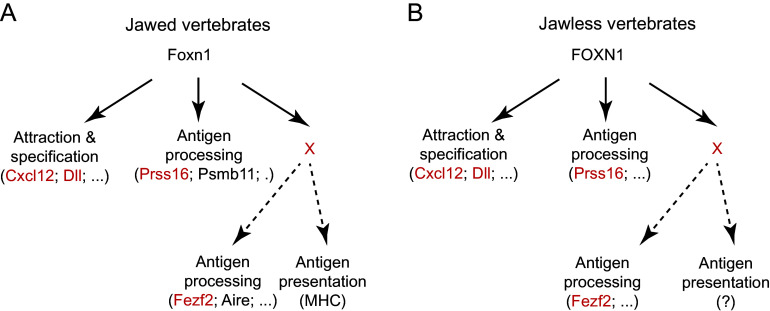
Structure of genetic networks underlying thymopoiesis in vertebrates. In jawed (*A*) and jawless (*B*) vertebrates, Foxn1 transcription factors regulate some target genes directly (solid arrows), whereas others are only indirectly targeted (dotted arrows) via additional transcription factors (designated X). The latter factors are presumed to be under control of Foxn1 but then activate genes required for certain aspects of antigen processing and presentation. Shared components are indicated in red font.

Our previous work demonstrated that in the mouse, combined expression of the Cxcl12 cytokine, and the Notch ligand Dll4 is sufficient to transform a *Foxn1*-deficient microenvironment into a lymphopoietically active niche in vivo; under these conditions, multipotent hematopoietic precursor cells enter the thymic rudiment, where they begin T cell differentiation that stalls at the CD4^+^CD8^+^ double-positive stage (28). This situation is reminiscent of the phenotype resulting from expression of the amphioxus *FOXN4* gene in the *Foxn1*-deficient mouse thymus; however, in this constellation, a small number of autoreactive T cells is generated (39). These findings suggest that although evolutionary ancestors of the vertebrate *Foxn1* genes are already “preadapted” to establish a lymphopoietic niche in the pharyngeal endoderm, they fail in orchestrating the essential quality control, indicating that the genetic underpinnings of early stages (supporting attraction of progenitors and induction of T cell fate) and late stages (supporting selection) of epithelial maturation are genetically separable. If so, the transcription factors encoded by vertebrate *Foxn1* genes must now be able to support these critical functions. A number of observations indicate that the evolutionarily conserved sequence specificity of the canonical DNA binding domain (46) and the C-terminal activation domain might suffice to activate target genes underlying the establishment of T cell identity in hematopoietic progenitor cells (39). Sequences outside of the canonical DNA binding domains, in particular the N-terminal regions of Foxn1, may provide the required interaction surfaces for additional cofactors (39) to expand the range of target genes including those required for antigen presentation, etc. (29), such as the serine protease Prss16, a vertebrate-specific gene (42) involved in the selection of CD4^+^ thymocytes (34). Based on studies in mice, MHC genes themselves are unlikely to be targets of Foxn1 (29), suggesting that other transcription factors fulfill this role downstream of the Foxn1-initiated differentiation programme. In Fig. 7, we schematically summarize these considerations to indicate the functional hierarchy of components of the thymic epithelial network. A key aspect of our hypothesis is that gene(s) X, which functions upstream of other components of the tolerance mechanism, is subject to control by the Foxn1 transcription factor. Note that X are not necessarily the same gene(s) in jawed and jawless vertebrates as there are examples of developmentally regulated genetic networks where the structure of the network is preserved whereas the identity of genes in the nodes is not (47). Nonetheless, the outcome of the replacement experiment suggests that gene(s) X governs further aspects of antigen processing, such as Fezf2 and Aire, and additional components of the antigen presentation pathway, such as MHC in the case of jawed vertebrates.

In conclusion, the results presented here begin to sketch out the conserved and unique components of thymopoiesis in jawed and jawless vertebrates (48).

## Materials and Methods

### Animals.

C57BL/6 mice were maintained in the Max Planck Institute of Immunobiology and Epigenetics. *Foxn1*^−/−^ mutant mice have been described previously (24). The lamprey FOXN1 cDNA sequences were inserted into pAHB14 (49) as *Not*I fragments; the *FOXN1* cDNA sequences used in the constructs were deposited in GenBank (*L. planeri*, PV987518; *G. australis*, PV987520; *C. wagneri*, PV987522); see *SI Appendix*, Fig. S3. To generate transgenic mice, constructs were linearized and injected into FVB pronuclei according to standard protocols. Transgenic founders were subsequently backcrossed to *Foxn1*-deficient mice on a C57BL/6J background. Mice were kept in the animal facility of the Max Planck Institute of Immunobiology and Epigenetics under specific pathogen-free conditions (14 h light, 10 h dark; temperature 22 ± 2 °C; relative humidity 55 ± 10%), and analyzed at the age of 4 to 6 wk. For identification of transgenes, the following primers were used for genotyping: *Foxn1:Mm_Foxn1*, SS40 (wild-type allele [5′-CTGTGAACTCAGCCATACTC]) + SS35 (wild-type and knockout allele [5′-TGCACCAAGCCTCTGCTGGGA]) + JBS003 (knockout allele [5′-TCGCCTTCTTGACGAGTTCT]), *Foxn1:Lp_FOXN1,* XAH163 (5′-GTCCCTAATCCGATGGCTAGCTC) + RM25 (5′- TTGAGTTGGCATCGCTCGTA); *Foxn1:Ga_FOXN1,* XAH163 (5′-GTCCCTAATCCGATGGCTAGCTC) + RM794 (5′- GACCGGTCTTTGAGCCGCTC); *Foxn1:Cw_FOXN1,* XAH163 (5′-GTCCCTAATCCGATGGCTAGCTC) + RM807 (5′- CTGACTTGACTTGAGCCCAG). Photos of mice were taken using the cameras from an iPhone 7 or iPhone SE.

All animal experiments were performed in accordance with the relevant guidelines and regulations, approved by the review committee of the Max Planck Institute of Immunobiology and Epigenetics and the Regierungspräsidium Freiburg, Germany (licenses AZ 35-9185.81/G-14/57). All strains are available from the corresponding author upon request, subject to standard material transfer agreements.

Ammocoete larvae of *G. australis* were collected from Lachlan River (New Norfolk, TAS, Australia) using backpack electrofishing equipment in March 2021. Twenty-six individuals were collected by Inland Fisheries Service Tasmania staff, and donated to this project. Larvae were either snap frozen in liquid nitrogen or fixed in 4% phosphate buffered formaldehyde solution. *C. wagneri* adults were collected from the Talar River (2 to 3 km from Ghaemshahr, Iran) in April 2021. Two sexually mature males that had been killed for another project were preserved in 70% ethanol, and fin clips from each individual were donated for use in the current project in August 2021.

### Source of Sequences.

Whole genome sequences for *G. australis* (SRR26437263) and *C. wagneri* (SRR26284203) are deposited in the Sequence Read Archive (BioProject accession number PRJNA1024071). Whole genome sequences for *L. planeri* have been described (50). Partial FOXN4 sequences were deposited in GenBank (*L. planeri*, PV987519; *G. australis*, PV987521; *C. wagneri*, PV987523).

### Immunohistochemistry.

Explanted thymic lobes were mounted and snap-frozen in optimal cutting temperature embedding compound. Tissue sections (10 μm) were cut using a cryostat and mounted onto precoated slides (Superfrost plus, Thermo Fisher Scientific). Slides were dried, washed in 1× PBS for 1 min followed by a 30-min blocking step using mouse immunoglobulin G (IgG) diluted 1:50 in PBS + 0.5% bovine serum albumin (BSA) + 0.2% Tween. K5 or K8 staining was performed with rabbit anti-K5 (BioLegend 905501 Clone Poly 19055, 1:500) and rat anti-K8 (Troma1, in-house, 1:200). As secondary antibodies, goat anti-rabbit Alexa Fluor 488 (A11008, Thermo Fisher Scientific, 1:500) and donkey anti-rat Cy3 (AB_2340668, Jackson ImmunoResearch, 1:500) were used. For ER-TR7 B220 staining, rat anti-mouse ERTR7 (BM4018, Acris, 1:200), donkey anti-rat IgG-Cy3 (712-166-153, Jackson, 1:500), rat anti-hu/mo B220-Alexa Fluor 488 (RA3-6B2, 53-0452-B2, Invitrogen, 1:100) were used. Sections were mounted with Fluoromount G before analysis (Apotome, Zeiss). For combined K5/K8/Aire staining, sections were dried, blocked, and stained with unlabeled primary antibodies as above and then stained with the secondary Abs goat anti-rabbit Alexa Fluor 633 (A21070, Invitrogen, 1:500), donkey anti-rat Cy3 (712-166-153, Jackson, 1:500). Sections were then blocked with rat IgG and subsequently stained with rat anti-mouse Aire Alexa Fluor 488 (5H12, eBioscience 14-5934-82, 1:200). Immunostained sections were imaged with Apotome2 AxioImager Z1 and Plan-Apochromat 10x/0,45 M27 as dry objective (Carl Zeiss AG, Oberkochen, Germany) running under ZEN Pro 3.2 blue software. Fluorophores were excited by an HXP 120 V lamp (Leistungselektronik Jena GmbH). For the detection of Alexa488 signal Zeiss Filter Set 38 HE was used, for Cy3 Zeiss Filter Set 43 HE, for DAPI Zeiss Filter Set 49 and for Alexa633 Zeiss Filter Set 50 He. Images were acquired with an AxioCam MRm black and white camera (Zeiss). All pictures were taken in Apotome mode. For capturing the whole thymus (*SI Appendix*, Fig. S2*A*) a Tile Scan was necessary, with 10% overlap of tiles. Afterward the images were processed and stitched with the help of ZEN blue software.

### RNA In Situ Hybridization.

Lamprey ammocoete larvae were fixed in 4% PFA and embedded in paraffin. Double in situ hybridization was carried out as follows. DIG- and fluorescein-labeled RNA antisense probes were simultaneously hybridized to RNA in tissue sections. The DIG-labeled probe was detected first, with an anti-DIG-POD antibody [1:300 dilution in MAB; 100 mM maleic acid, pH 7.5, 150 mM NaCl, 2 mM Levamisol, 1% blocking reagent (Roche), 0.1% Tween 20] and revealed by Cy3 fluorescence, using the Tyramide Signal Amplification Plus system (AkoyaBioscience). The sections were washed several times with PBS; the fluorescein-labeled probe was detected by a peroxidase-conjugated anti-Fluorescein-POD (1:300 dilution in MAB) and revealed by Cy5 fluorescence. Sequence coordinates in GenBank accession numbers for the probes were as follows: a) *L. planeri*: *CDA2*, nucleotides 15412017-15411834 joined to 15411064-15410909 in OZ078335.2; *VLRB*, nucleotides 1-878 in FJ187756.1. *DLL*, nucleotides 12411475 to 12411974 in OZ078353.2; *FOXN1*, join nucleotides 7302908 to 7302709, 7300794 to 7300496 in OZ078329.2; *FEZF2*, nucleotides 9652765 to 9653264 in OZ078357.2; *CXCL12*, join nucleotides 1853787 to 1853671, 1857448 to 1857213 in OZ078332.2; PRSS16, join nucleotides 8971332 to 8971216, 8970697 to 8970539, 8968890 to 8968826, 8967453 to 8967385, 8966196 to 8966121 in OZ078372.2. b) *G. australis*: *CDA2*, join nucleotides 11583813-11583447 and 11582538-11582330 in JAZEWU010000007.1; *VLRB*, nucleotides 408918-408378 in JAZEWU010000044.1.

A quantitative analysis of positive cells beyond a qualitative recording in the thymoid is hampered by the complex histological structure of the lamprey gill basket. Images were acquired on Zeiss microscopes (Axioplan 2) equipped with an Mrc5 camera and Zeiss AxioZoom V.16; in some figure panels, fluorescent signals were converted to false color for better visualization.

### Flow Cytometry.

Single-cell suspensions of TECs for preparative flow cytometry were obtained as described (13). Analytical flow cytometry was performed for TECs as follows: anti-CD45 (30-F11), conjugated with PE/Cy7 (1:2,000 dilution, BioLegend); anti-EpCAM (G8.8), conjugated with APC (1:1,000 dilution, BioLegend); anti-Ly51 (alias BP-1; 6C3), conjugated with biotin (1:300 dilution, eBioscience) in combination with streptavidin, conjugated with eFluor 450 (1:1,000 dilution, eBioscience); UEA1, conjugated with FITC (1:2,000 dilution, Vector Labs). When analysis of hematopoietic fractions was desired from thymic lobes and lymph nodes, cell suspensions were prepared in parallel by mechanical liberation, best achieved by gently pressing tissues through 40 μm sieves. Cell surface staining was carried out using anti-CD45 (30-F11), conjugated with PE/Cy7 (1:2,000 dilution, BioLegend); anti-CD4 (GK1.5), conjugated with FITC (1:1,000 dilution, BioLegend); anti-CD8a (53-6.7), conjugated with APC (1:800 dilution, eBioscience); anti-CD19 (eBio1D3), conjugated with PerCP/Cy5.5 (1:500 dilution, eBioscience) or PE/Cy7 (1:1,000 dilution, eBioscience); anti-B220 (alias CD45R; RA3-6B2), conjugated with biotin (1:200 dilution, eBioscience); anti-IgM (II/4.1), conjugated with PE (1:300 dilution, eBioscience); anti-CD93 (alias C1qRp; AA4.1), conjugated with APC (1:300 dilution, eBioscience); streptavidin conjugated with eFluor 450 or FITC (1:1,000 dilution, eBioscience). For analysis of DN thymocytes, a lineage cocktail comprising anti-B220 (alias CD45R; RA3-6B2), conjugated with biotin (1:200 dilution, eBioscience); anti-TCRgd (GL3), conjugated with biotin (1:100 dilution, eBioscience); anti-Nk1.1 (PK136), conjugated with biotin (1:500 dilution, eBioscience); anti-Cd11b (M1/70), conjugated with biotin (1:200 dilution, eBioscience); anti-Cd11c (HL3), conjugated with biotin (1:100 dilution, BD Biosciences) was used. Flow cytometry experiments were evaluated using FACSDiva (8.0.2) and FlowJo (9.3.1) software. The relevant gating strategies are shown in *SI Appendix*, Fig. S9.

### Sequence Alignments.

Protein sequences were aligned using the Clustal W algorithm implemented in the DNAStar Lasergene suite of programmes (https://www.dnastar.com) or by Clustal Omega (51).

### Bulk RNAseq Data Availability and Processing.

Bulk RNAseq samples for wild type TECs and TECs reconstituted with *L. planeri FOXN1* have been deposited with the European Nucleotide Archive (accession numbers ERS27074366- ERS27074372, project PRJEB100831). Adapter sequences were trimmed from raw reads with cutadapt v4.9 (52).

For investigation of *Foxn1* splice patterns, the reference genome and gene annotations were sourced from Ensembl (GRCm39, Ensembl release 113). The trimmed reads were aligned to the reference with STAR v2.7.11b (53), and coverage over the mouse *Foxn1* locus was visualized with Gviz v1.46.1 (54).

Differential gene expression analysis and analysis of canonical and mimetic TEC signatures was carried out as described previously (13). Reads were aligned to the mouse reference genome GRCm38 with HISAT2 v2.1.0 (55). Gene-level counts were generated with featureCounts v1.6.1 (56). Differential expression analysis was performed using voom (57) and limma (58, 59). The t-statistic per gene was used as input for the competitive gene set test cameraPR (60); the Benjamini–Hochberg adjusted *P* values of cameraPR were log_10_ transformed. For upregulated signatures, the resulting value was multiplied with −1, so that positive and negative values indicate an upward or downward direction of change, respectively.

### Statistical Analysis.

To compare cell counts, count ratios, and proportions between conditions, generalized linear models with the respective dependent variables and genotype and transgenic status as predictors were fitted with R (v4.4.3), using the glmmTMB package (v1.1.11; ref. 61). For count data, the models used a negative binomial distribution with a log link function; for count ratios, an offset of log(denominator) was incorporated into the model; for proportions, a beta distribution with a logit link function was used. Effect sizes, their CI and *P* values of contrasts between groups were determined with the emmeans package (v1.11.1).

## Supplementary Material

Appendix 01 (PDF)

## Data Availability

DNA sequences data have been deposited in Genbank European Nucleotide Archive: PV987518-PV987523 (62–67), SRR26284203 (68), ERS27074369-ERS27074372 (69), and SRR26437263 (70). All other data are included in the manuscript and/or *SI Appendix*.
