# Supplementary material for: Lamprey FOXN1 rescues the block of thymic epithelial cell development in the mouse Foxn1-deficient thymic rudiment
Source: Proc Natl Acad Sci U S A. 2025 Nov 25;122(48):e2520664122. doi: 10.1073/pnas.2520664122 (PMC12685072; doi:10.1073/pnas.2520664122)
Supplement: Supplementary file 1 — Appendix 01 (PDF) [file pnas.2520664122.sapp.pdf]

PNAS

Supporting Information for

Lamprey *FOXN1* rescues the block of thymic epithelial cell development in the mouse *Foxn1*-deficient thymic rudiment

Ryo Morimoto, Gaoqun Zhang, Oliver S. Thomas, Margaret Docker, Jonah L. Yick, Floriaan Devloo-Delva, Jeremy Swann, Dagmar Diekhoff, Thomas Boehm

This PDF file includes:

Figures S1 to S9

References

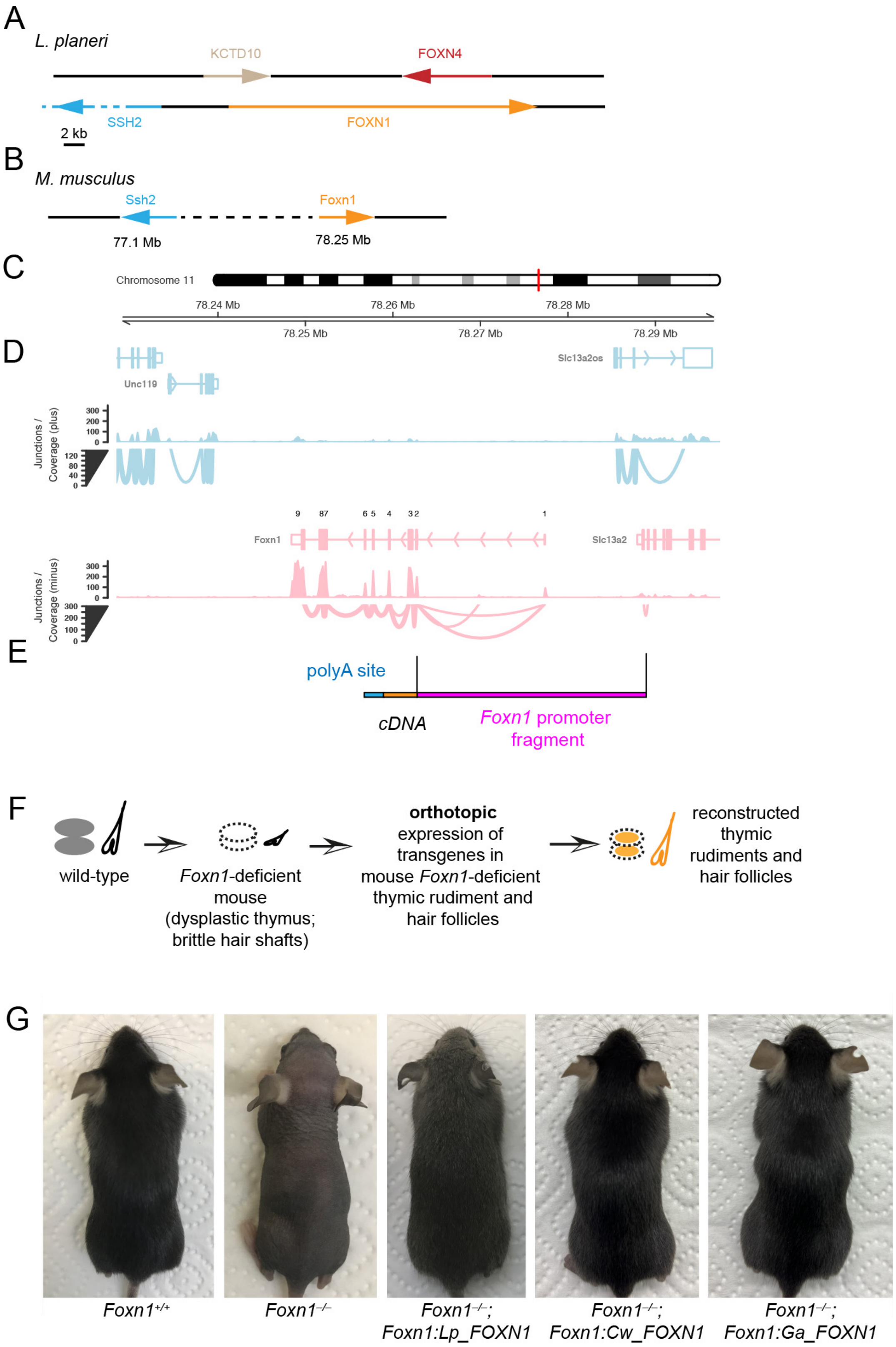
A
L. planeri
KCTD10
FOXN4
SSH2
FOXN1
2 kb
B
M. musculus
Ssh2
Foxn1
77.1 Mb
78.25 Mb
C
Chromosome 11
78.24 Mb
78.26 Mb
78.28 Mb
78.25 Mb
78.27 Mb
78.29 Mb
D
Unc119
Slc13a2os
Junctions / Coverage (plus)
Foxn1
Slc13a2
Junctions / Coverage (minus)
E
polyA site
cDNA
Foxn1 promoter fragment
F
wild-type
Foxn1-deficient mouse (dysplastic thymus; brittle hair shafts)
orthotopic expression of transgenes in mouse Foxn1-deficient thymic rudiment and hair follicles
reconstructed thymic rudiments and hair follicles
G
Foxn1+/+
Foxn1−/−
Foxn1−/−; Foxn1:Lp_FOXN1
Foxn1−/−; Foxn1:Cw_FOXN1
Foxn1−/−; Foxn1:Ga_FOXN1

Fig. S1. Experimental strategy of *Foxn1* replacement. (*A*) Syntenic context of lamprey *Foxn4* and *Foxn1* genes; arrows indicate the direction of transcription. The lamprey genome encodes two members of the *Foxn1/4* gene family; the *Foxn4* gene is orthologous to the ancient metazoan member of this family, whereas the *Foxn1* gene represents a vertebrate-specific paralog (*1*). The *FOXN1* gene is expressed in the thymoid (Fig. 1). (*B*) Evolutionary conservation of the overall *Ssh2* and *Foxn1* synteny; the approximate positions of the two genes on mouse chromosome 11 are indicated. (*C*) Detailed view of the mouse *Foxn1* locus with the two flanking genes indicated; exons are boxed. (*D*) Alignment of mouse *Foxn1* transcripts in TECs indicating the preferential use of the upstream promoter (adjacent to the noncoding exon 1a); the downstream promotor, upstream of exon 1b is preferentially used in keratinocytes of the hair shaft. (*E*) Schematic of the transgenic construct. A large genomic fragment (pink box) encompassing the 3′-end of the *Slc13a2* gene (providing a polyA site to prevent read-through into the *Foxn1* region), the two *Foxn1*-associated promotors, and the non-coding nucleotides of exon 2 are fused to the relevant cDNA fragment (yellow box), followed by three polyA sites (blue box) (*2*). (*F*) Experimental strategy used in this study. In *Foxn1*-deficient mice, the thymic rudiment is dysplastic and fails to attract haematopoietic progenitor cells; the hair shafts are incompletely keratinized and owing to their brittle nature easily break, giving the appearance of a "nude" skin. Orthotopic expression of *FOXN1* genes may restore failing TEC and hair shaft differentiation. (*G*) Hair coat structure of wild type and transgenic mice. Shown are wild type (*Foxn1*$^{+/+}$), *Foxn1*-deficient (*Foxn1*$^{-/-}$), and mice expressing the *FOXN1* genes of the three lamprey species studied here (*L. planeri*, *Foxn1:Lp_FOXN1*; *C. wagneri*, *Foxn1:Cw_FOXN1*; *G. australis*, *Foxn1:Ga_FOXN1*) on the *Foxn1*-deficient background.

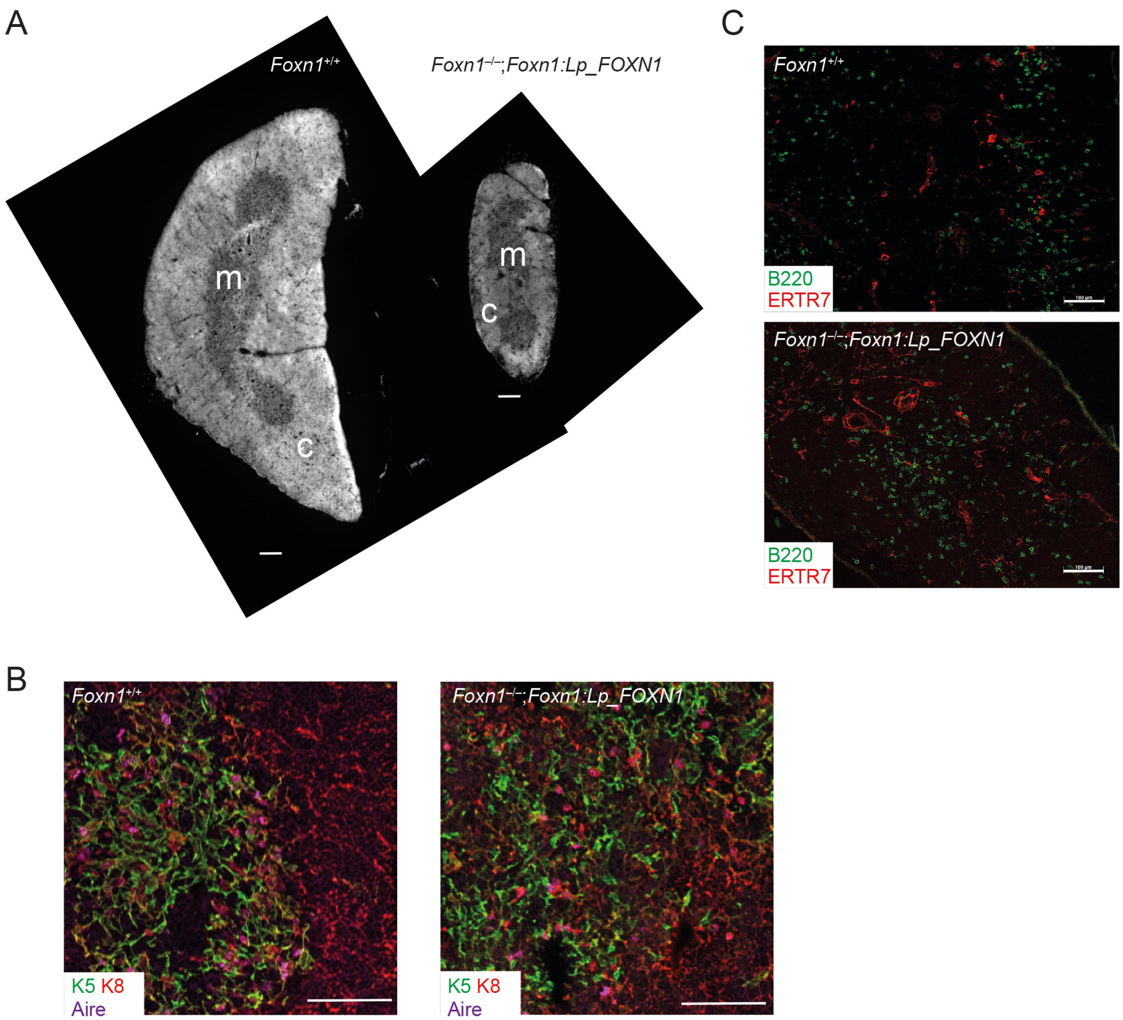

Fig. 2. Characteristics of the thymus in *Foxn1*$^{-/-}$;*Foxn1:Lp_FOXN1* transgenic mice. (*A*) Representative sections of thymic lobes stained with DAPI. m, medulla; c, cortex. Note the size difference between the wild type and the transgenic thymus. Scale bars, 0.2mm. (*B*) Immunohistology using anti-keratin antibodies (K5, green; K8, red), an antibody against the autoimmune regulator Aire (magenta). Scale bars, 0.1mm.
(*C*) Immunohistochemical analysis using antibodies against ERTR7 (a marker of thymic fibroblasts), and B220, a B cell-specific isoform of CD45. Scale bars 0.1mm.

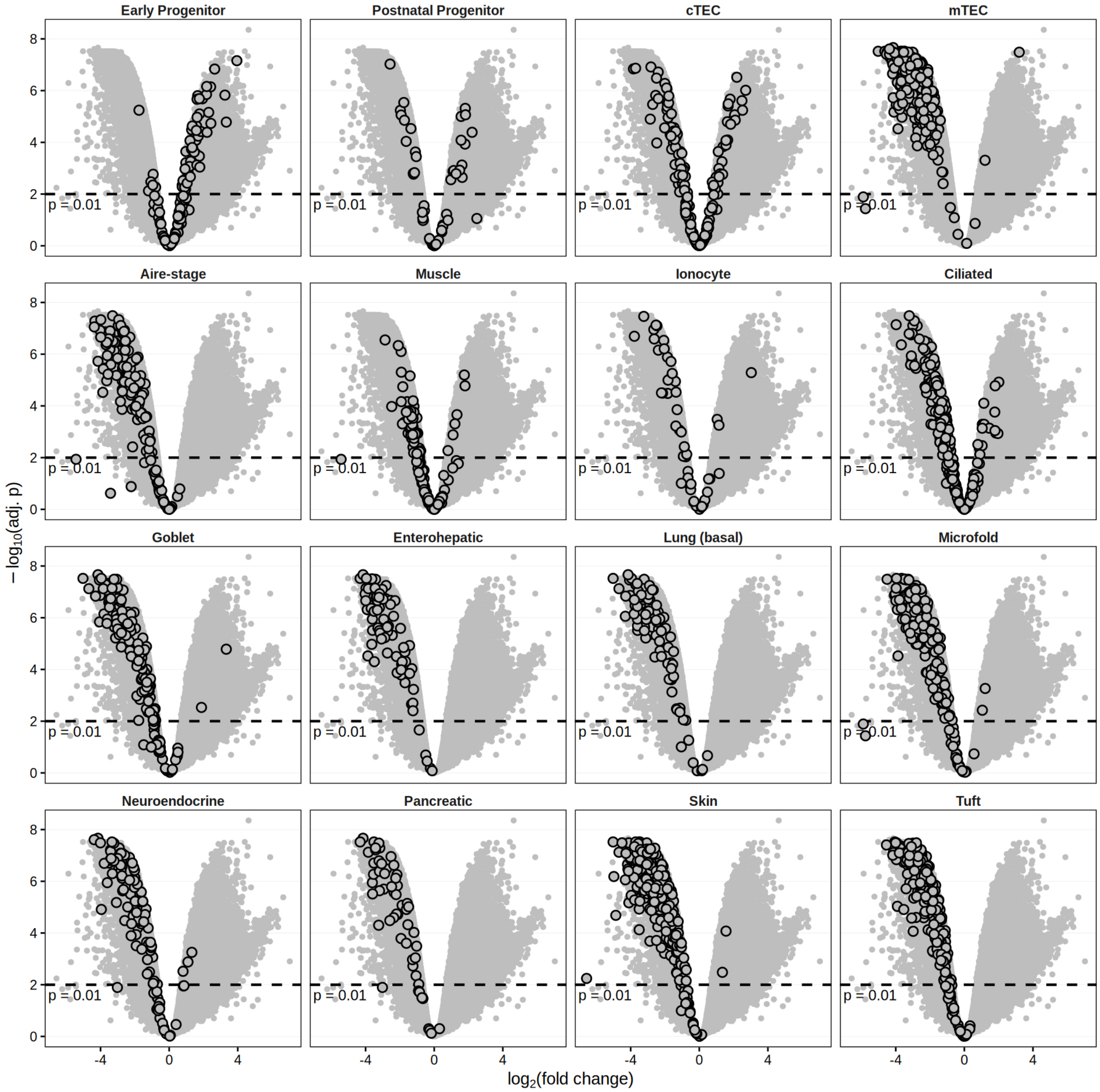

Supplementary Figure 3. Differential gene expression analysis between wild type and *L. planeri FOXN1* transgenic TECs. Volcano plots showing the results of differential expression analysis between wild type (n=3) and *L. planeri FOXN1* transgenic (n=4) mice. In each panel, genes which are part of the respective TEC signatures are highlighted.

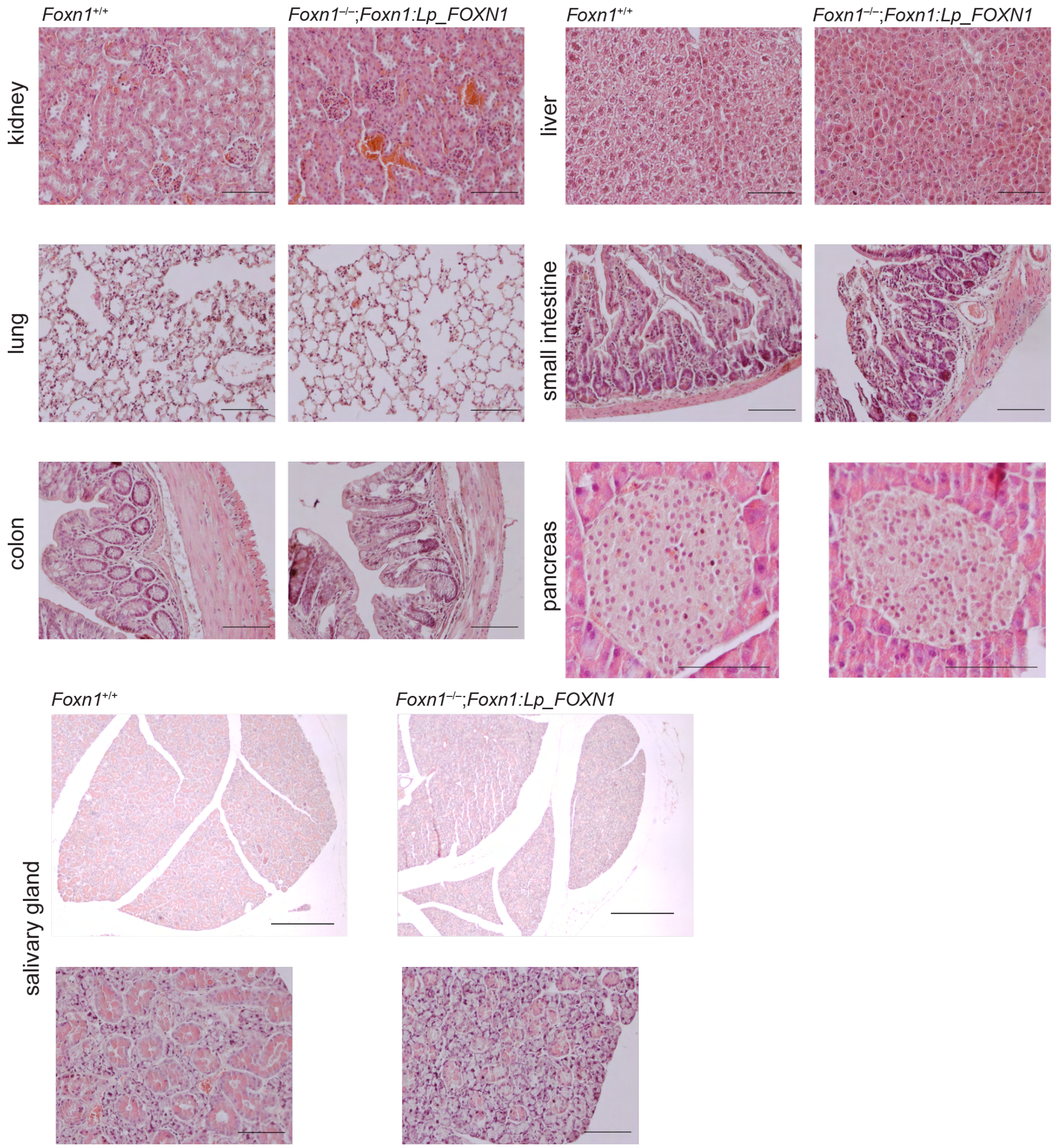

Supplementary Figure 4. Absence of tissue infiltrations in *L. planeri FOXN1* transgenic mice. Tissue sections of wild type (*Foxn1*$^{+/+}$) and *Lp_FOXN1* transgenic mice (*Foxn1*$^{-/-}$; *Lp_FOXN1*) were stained with haematoxylin/eosin. All scale bars represent 0.1mm, with the exception of the top panels shown for salivary glands, where they represent 0.5mm.

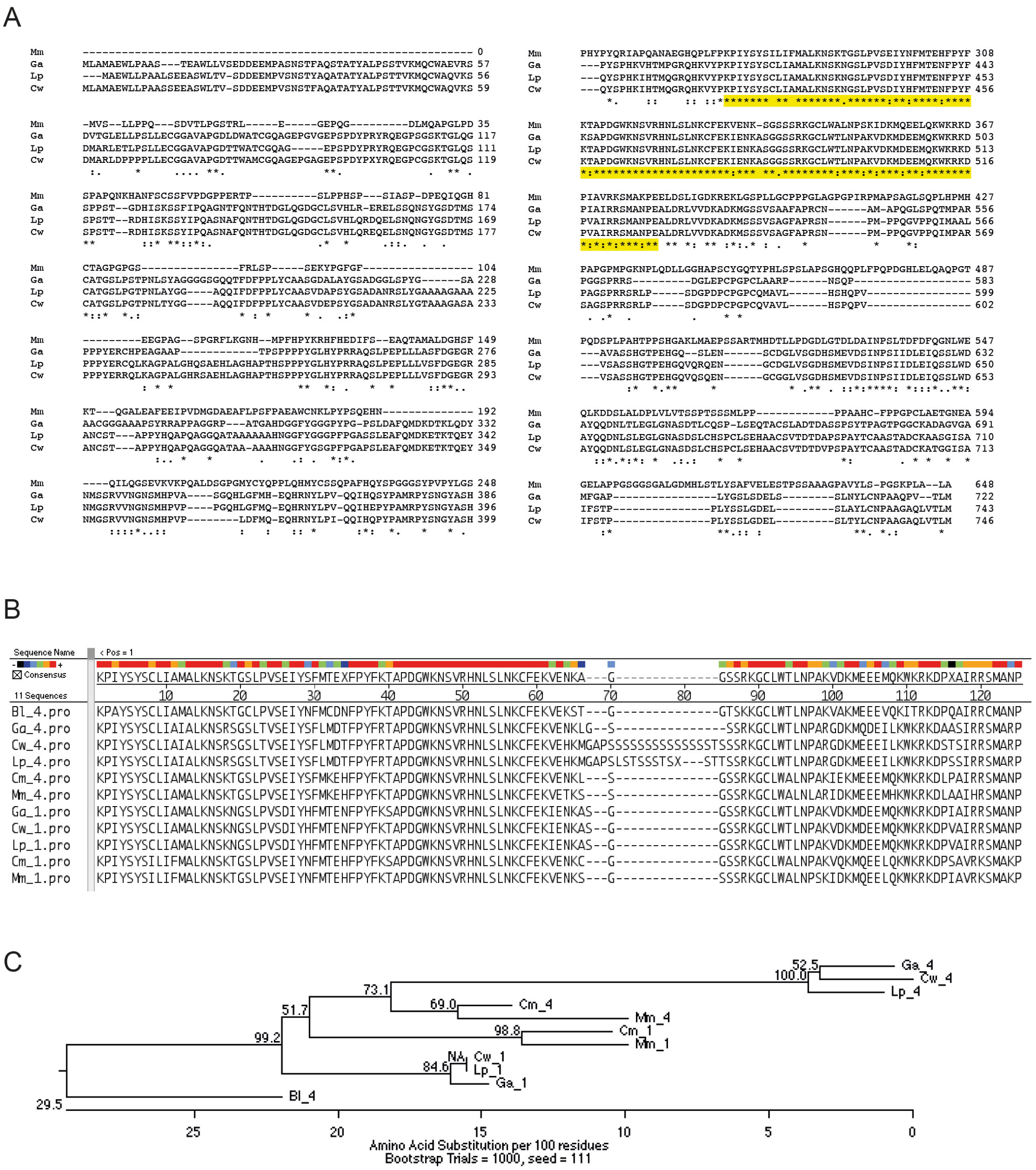

Fig. S5. Sequence signatures of Foxn1/4 proteins. (*A*) Multiple sequence alignment of Foxn1 protein sequences of the indicated species; the DNA binding domain is highlighted in yellow (see panel B). Mm, *Mus musculus*. Ga, *Geotria australis*; Lp, *Lampetra planeri*; Cw, *Caspiomyzon wagneri.* (*B*) Multiple sequence alignment of deduced protein sequence of DNA binding domains of Foxn1 and Foxn4 proteins for different species. Source of sequences, GenBank accession numbers: Bl, *Branchiostoma lanceolatum* FOXN4, XP_035692028; Ga_1, *Geotria australis* FOXN1, PV987520; Ga_4, *Geotria australis* FOXN4, PV987521; Cw_1, *Caspiomyzon wagneri* FOXN1, PV987522; Cw_4, *Caspiomyzon wagneri* FOXN4, PV987523; Lp_1, *Lampetra planeri* FOXN1, PV987518;

Lp_4, *Lampetra planeri* FOXN4, PV987519; Cm_1, *Callorhinchus milii* Foxn1, ACN53914 ; Cm_4, *Callorhinchus milii* Foxn4, NP_001279572; Mm_1, *Mus musculus* Foxn1, XP_006532329; Mm_4, *Mus musculus* Foxn4, NP_683737. *(C*) Derived phylogenetic tree (Clustal W) indicating the sequence relationships of the DBDs, with bootstrap values indicated. Species abbreviations as in panel B.

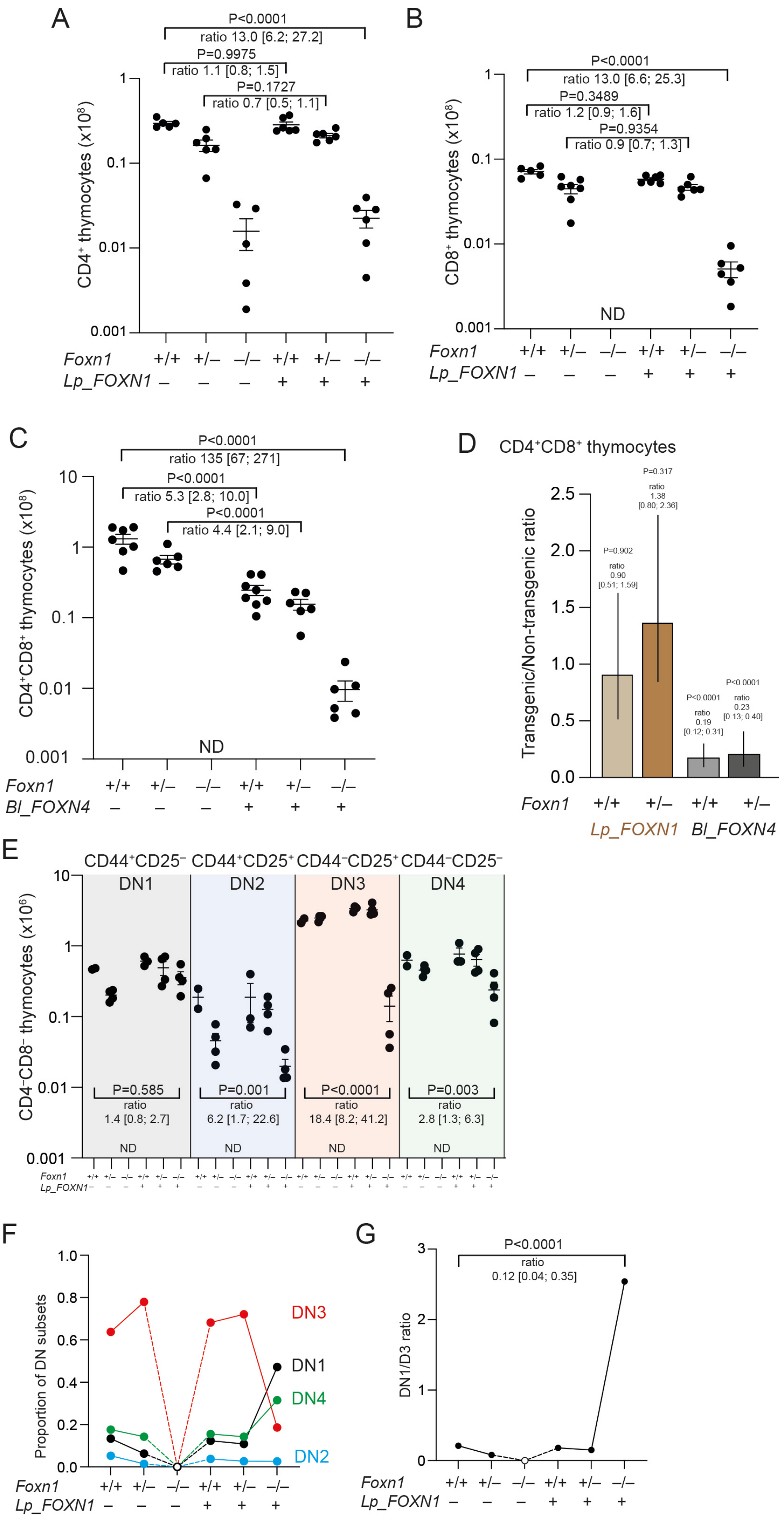
A
P<0.0001
ratio 13.0 [6.2; 27.2]
P=0.9975
ratio 1.1 [0.8; 1.5]
P=0.1727
ratio 0.7 [0.5; 1.1]
CD4⁺ thymocytes (x10⁸)
1
0.1
0.01
0.001
Foxn1 +/+ +/– –/– +/+ +/– –/–
Lp_FOXN1 – – – + + +
B
P<0.0001
ratio 13.0 [6.6; 25.3]
P=0.3489
ratio 1.2 [0.9; 1.6]
P=0.9354
ratio 0.9 [0.7; 1.3]
CD8⁺ thymocytes (x10⁸)
ND
Foxn1 +/+ +/– –/– +/+ +/– –/–
Lp_FOXN1 – – – + + +
C
P<0.0001
ratio 135 [67; 271]
P<0.0001
ratio 5.3 [2.8; 10.0]
P<0.0001
ratio 4.4 [2.1; 9.0]
CD4⁺CD8⁺ thymocytes (x10⁸)
10
1
0.1
0.01
0.001
ND
Foxn1 +/+ +/– –/– +/+ +/– –/–
Bl_FOXN4 – – – + + +
D
CD4⁺CD8⁺ thymocytes
Transgenic/Non-transgenic ratio
P=0.902
ratio
0.90
[0.51; 1.59]
P=0.317
ratio
1.38
[0.80; 2.36]
P<0.0001
ratio
0.19
[0.12; 0.31]
P<0.0001
ratio
0.23
[0.13; 0.40]
Foxn1 +/+ +/– +/+ +/–
Lp_FOXN1 Bl_FOXN4
E
CD44⁺CD25⁻ CD44⁺CD25⁺ CD44⁻CD25⁺ CD44⁻CD25⁻
DN1 DN2 DN3 DN4
CD4⁻CD8⁻ thymocytes (x10⁸)
P=0.585
ratio
1.4 [0.8; 2.7]
P=0.001
ratio
6.2 [1.7; 22.6]
P<0.0001
ratio
18.4 [8.2; 41.2]
P=0.003
ratio
2.8 [1.3; 6.3]
ND
Foxn1
Lp_FOXN1
F
Proportion of DN subsets
DN3
DN1
DN4
DN2
Foxn1 +/+ +/– –/– +/+ +/– –/–
Lp_FOXN1 – – – + + +
G
P<0.0001
ratio
0.12 [0.04; 0.35]
DN1/D3 ratio
Foxn1 +/+ +/– –/– +/+ +/– –/–
Lp_FOXN1 – – – + + +

Fig. S6. Characterization of thymopoiesis in *FOXN1* transgenic mice. (*A*) Number of $CD4^+$ single-positive thymocytes in *Foxn1:Lp_FOXN1* transgenic mice. (*B*) Number of $CD8^+$ single-positive thymocytes in *Foxn1:Lp_FOXN1* transgenic mice. No $CD8^+$ single-positive thymocytes are detectable in *Foxn1*$^{-/-}$ mice, denoted as ND. (*C*) Number of $CD4^+CD8^+$ double-positive (DP) thymocytes in *Foxn1:Bl_FOXN4* transgenic mice demonstrating a strong dominant-negative effect of the transgene. No DP thymocytes are detectable in *Foxn1*$^{-/-}$ mice (ND) (*D*) Contrasting effects of *Foxn1:Lp_FOXN1* and *Foxn1:Bl_FOXN4* transgene expression on T cell development. The expression of the *Foxn1:Lp_FOXN1* transgene in the *Foxn1*$^{+/+}$ and *Foxn1*$^{+/-}$ heterozygous backgrounds has minimal effect (Fig. 2B), whereas the expression of the *Foxn1:Bl_FOXN4* transgene suppresses T cell development (see panel (*C*) of this Figure). (*E*) Absolute numbers of early thymocytes ($CD4^-CD8^-$ double-negative thymocytes [DN]) in *Foxn1:Lp_FOXN1* transgenic mice. (*F*) Proportion of DN subsets in *Foxn1:Lp_FOXN1* transgenic mice. (*G*) Ratio of DN1 and DN3 subsets in *Foxn1:Lp_FOXN1* transgenic mice.
For all panels, the genotypes of mice are indicated at the bottom. ND denotes the absence of cells with the indicated phenotype; in panels F and G, no ratio could be calculated for the *Foxn1*$^{-/-}$ genotype (denoted by open circle). For panels A, B, C, and E, the sample mean ± SEM is shown. For panels A- E, G, the P values and estimated ratios of cell numbers for the indicated comparisons are indicated, with 95% confidence intervals in square brackets.

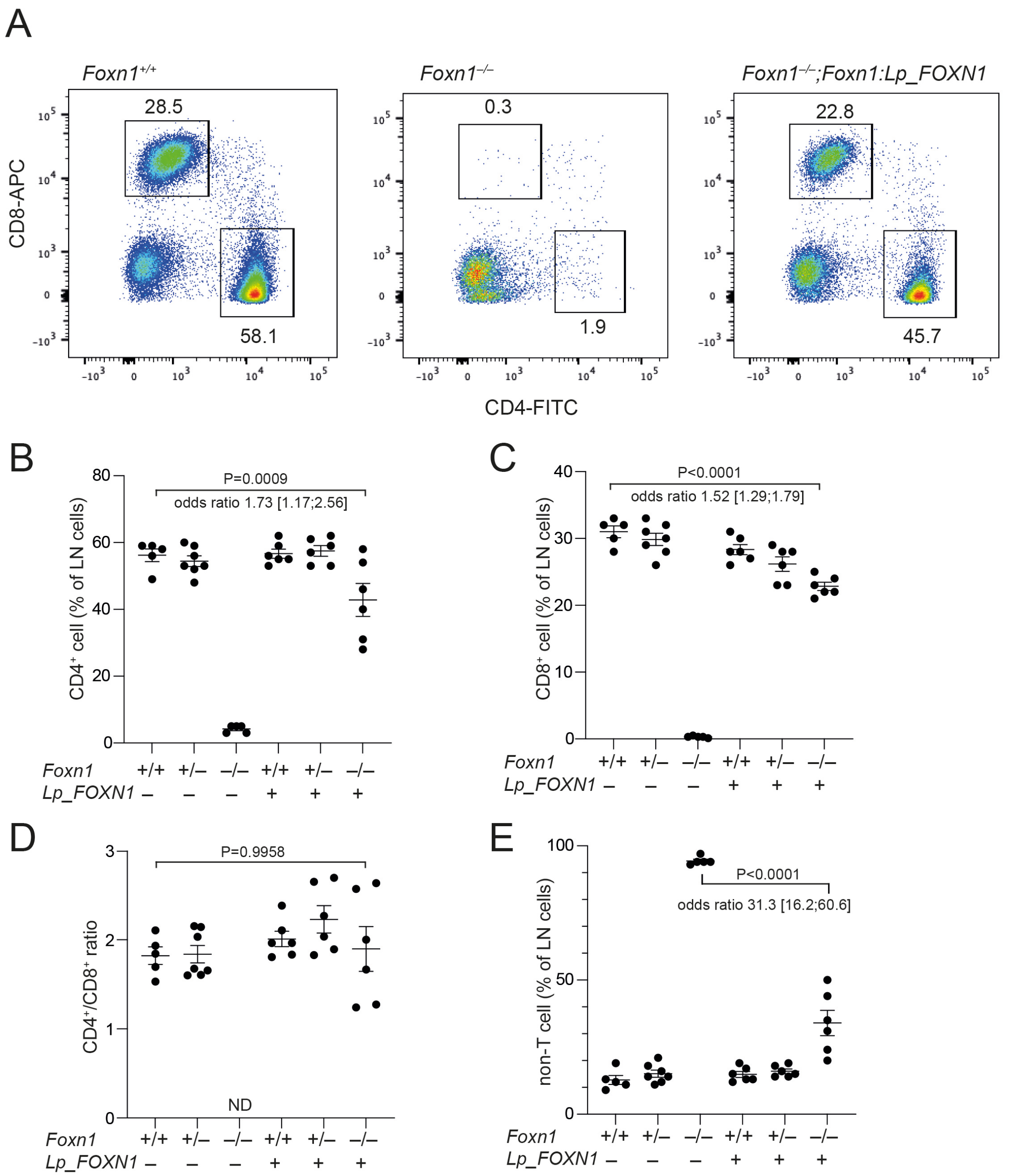

Fig. S7. Characterization of the peripheral T cell compartment in *Foxn1:Lp_FOXN1* transgenic mice. (*A*) Representative flow cytometric profiles of $CD45^+$ cells resolved into $CD4^+$ and $CD8^+$ subsets. (*B*) Percentage of $CD4^+$ single-positive T cells in lymph nodes. (*C*) Percentage of $CD8^+$ single-positive T cells in lymph nodes. (*D*) Ratio of $CD4^+$ and $CD8^+$ single-positive T cells in lymph nodes. (*E*) Percentage of non-T cells in lymph nodes. For all panels, the genotypes of mice are indicated at the bottom. ND denotes the absence of cells with the indicated phenotype. For panels B, C, E, the P values and odds

ratios of proportions for the indicated comparisons are indicated, with 95% confidence intervals in square brackets.

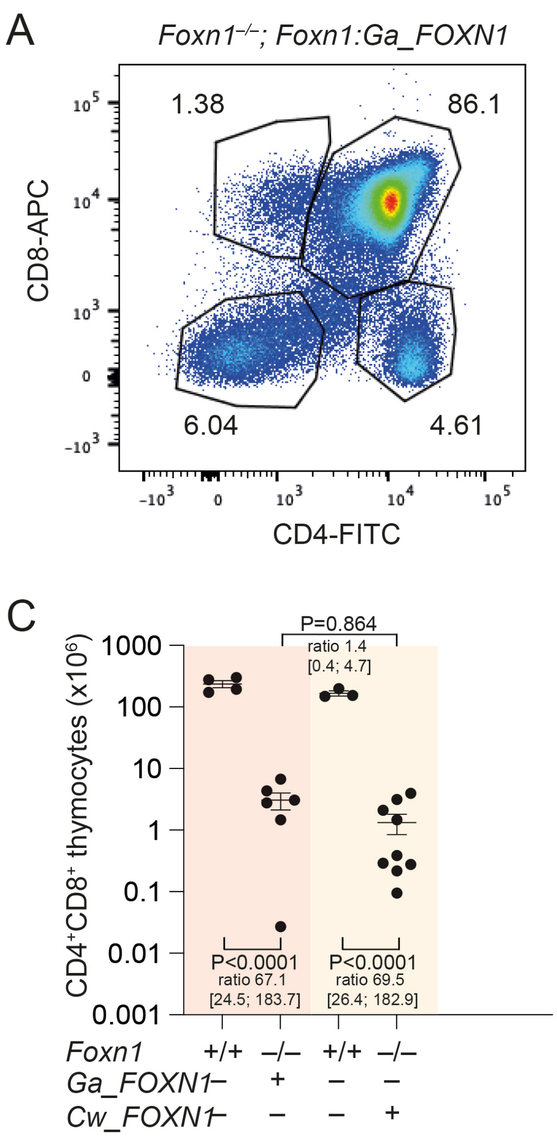

Fig. S8. Characterization of thymopoiesis and the peripheral T cell compartment of transgenic mice. (*A,B*) Representative flow cytometric profiles of CD45$^+$ thymocytes resolved into CD4$^+$ and CD8$^+$ subsets. The genotypes of mice are indicated. (*C*) Absolute numbers of CD4$^+$CD8$^+$ double-positive T cells in the thymus. The genotypes of mice are indicated at the bottom, and are shown for the two pairs of wild type and transgenic mice. Ga, *Geotria australis*; Cw, *Caspiomyzon wagneri*. For panel C, the P values and ratios of cell numbers for the indicated comparisons are indicated, with 95% confidence intervals in square brackets.

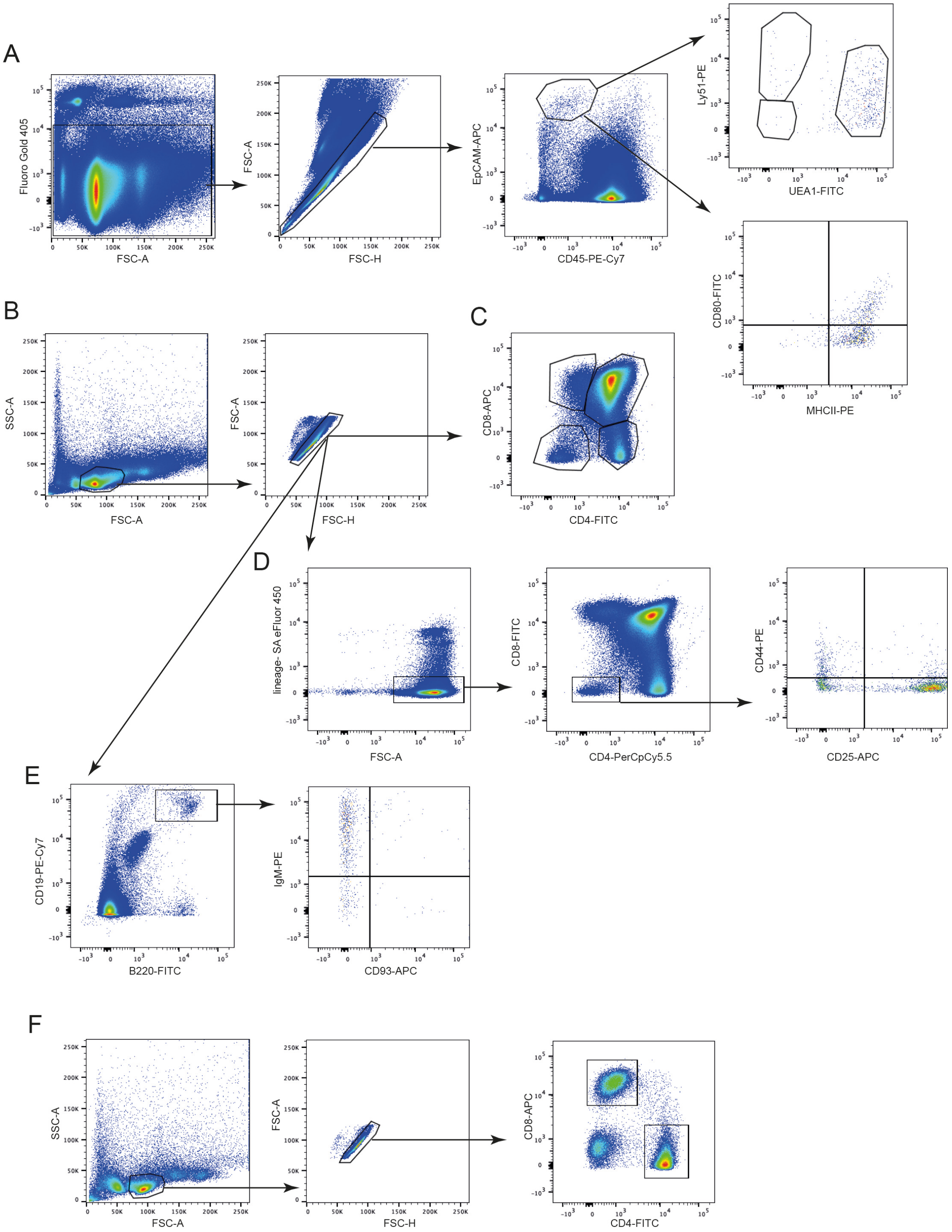

Fig. S9. Gating strategies for the flow cytometric analyses used. (*A*) Characterization of thymic epithelial cells (TECs) as EpCAM$^+$CD45$^-$ cells. This subset can be differentiated either into cortical (Ly51$^+$;UEA1$^-$), medullary (Ly51$^-$;UEA1$^+$), and undifferentiated (Ly51$^-$;UEA1$^-$) cells or into mature mTECs (MHCII$^+$;CD80$^+$). (*B-E*) Single haematopoietic cells (*B*) can be characterized by their expression of CD4 and CD8

coreceptors (*C*), $CD4^{-}CD8^{-}$ double-negative subsets using CD44 and CD25 expression patterns (*D*), or for B cell subsets using IgM and CD93 expression patterns (*E*). (*F*) Single lymph node T cells can be characterized by their expression of CD4 and CD8 coreceptors.

References

1. B. Bajoghli, et al., Evolution of genetic networks underlying the emergence of thymopoiesis in vertebrates. *Cell* **138**, 186-197 (2009).

2. C. C. Bleul, T. Boehm, BMP signaling is required for normal thymus development. *J. Immunol.* **175**, 5213-5221 (2005).
